# The rise of angiosperm-dominated herbaceous floras: Insights from Ranunculaceae

**DOI:** 10.1038/srep27259

**Published:** 2016-06-02

**Authors:** Wei Wang, Li Lin, Xiao-Guo Xiang, Rosa del C. Ortiz, Yang Liu, Kun-Li Xiang, Sheng-Xiang Yu, Yao-Wu Xing, Zhi-Duan Chen

**Affiliations:** 1 State Key Laboratory of Systematic and Evolutionary Botany, Institute of Botany, Chinese Academy of Sciences, Beijing 100093, China; 2 Missouri Botanical Garden, P.O. Box 299, St. Louis, Missouri 63166-0299, USA; 3 Department of Ecology and Evolutionary Biology, University of Connecticut, Storrs, CT 06269-3043, USA; 4 Field Museum of Natural History, 1400 S Lake Shore Drive, Chicago, IL 60605, USA

## Abstract

The rise of angiosperms has been regarded as a trigger for the Cretaceous revolution of terrestrial ecosystems. However, the timeframe of the rise angiosperm-dominated herbaceous floras (ADHFs) is lacking. Here, we used the buttercup family (Ranunculaceae) as a proxy to provide insights into the rise of ADHFs. An integration of phylogenetic, molecular dating, ancestral state inferring, and diversification analytical methods was used to infer the early evolutionary history of Ranunculaceae. We found that Ranunculaceae became differentiated in forests between about 108–90 Ma. Diversification rates markedly elevated during the Campanian, mainly resulted from the rapid divergence of the non-forest lineages, but did not change across the Cretaceous-Paleogene boundary. Our data for Ranunculaceae indicate that forest-dwelling ADHFs may have appeared almost simultaneously with angiosperm-dominated forests during the mid-Cretaceous, whereas non-forest ADHFs arose later, by the end of the Cretaceous terrestrial revolution. Furthermore, ADHFs were relatively unaffected by the Cretaceous-Paleogene mass extinction.

On the reorganization and modernization of terrestrial ecosystems, two broad-scale events in the Cretaceous period have had a crucial effect: Cretaceous Terrestrial Revolution (KTR) and Cretaceous-Paleogene (K-Pg) mass extinction^1^,^2^,^3^. The KTR, from 125 to 80 million years ago (Ma), is marked by the fact that terrestrial diversity vastly surpassed the diversity in the seas for the first time^4^ and that the angiosperm component of floras increased from 0 to 80%^3^. The compiled data on plant fossils indicate that angiosperms experienced an explosive radiation and for the first time achieved widespread floristic dominance between about 115 to 90 Ma^5^,^6^. During this period, angiosperm venation as a significant evolutionary innovation experienced a rapid and sharp increase in the vein length density^7^,^8^, leading to that the photosynthetic and transpiration capacities of angiosperms greatly increased and exceeded those of non-angiosperms^8^,^9^,^10^. The rapid rise and expansion of angiosperms has thereby strongly altered productivity on land, climate, and the global hydrological cycle^2^,^4^,^11^,^12^,^13^. Many studies have supported a causal link between the rapid rise of angiosperms and the Cretaceous pulses of increased diversification of various land-dwelling organisms, including various non-angiosperms^14^,^15^, insects^16^,^17^, and tetrapods^3^,^18^. Thus, the KTR is also referred as the angiosperm revolution^19^, and the rapid rise of angiosperms has been regarded as the putative trigger of the KTR^15^.

Angiosperms consist of woody and herbaceous plants. Woody angiosperms, predominantly represented by trees and shrubs, are the conspicuous elements of forests in temperate and tropical regions, whereas herbaceous angiosperms dwell in angiosperm-dominated forests or are the dominant plant life in open vegetations of middle to high latitudes or altitudes. The timing of the inferred radiation of rosids, which include many important trees of extant temperate and tropical forests, suggests that angiosperm-dominated forests rapidly arose around 108 to 83 Ma^20^, which highly coincides with the timing of the explosive radiation of angiosperms. However, very little is known about the rise of angiosperm-dominated herbaceous floras (ADHFs) to date, neither in angiosperm-dominated forests nor in open vegetations.

At the end of the Cretaceous, ~66 Ma, the K-Pg extinction, is the latest of the “big five” mass extinctions in the history of life^21^,^22^, and is regarded as the final step in the reorganization of modern ecosystems^3^. The latest mass extinction resulted from the Chicxulub asteroid impact and Deccan volcanism^23^,^24^, when dust and sulphate aerosols shot into the atmosphere by the impact and volcanism would have darkened the skies, causing a shutdown of photosynthesis and subsequent collapse of ecosystems^25^,^26^. Diversification rate analyses indicate that Menispermaceae experienced a sudden increase of lineage accumulation near the K-Pg boundary^27^. As an important representative of liana families in tropical rainforests, this study on Menispermaceae implies that tropical rainforests may have been dramatically destructed at the K-Pg boundary^27^. However, the effect of the K-Pg mass extinction on ADHFs remains to be addressed.

Herb fossil records are few and can not provide an accurate temporal framework for the evolution of ADHFs in the Cretaceous. Newly developed model-based methods that allow estimation of variation in diversification rates among lineages^28^,^29^ have become powerful tools for assessing diversification dynamics of groups with a poor fossil record. In this paper we investigate the rise of ADHFs in relation to the KTR and K-Pg mass extinction events by examining the early diversification of the herbaceous angiosperm family Ranunculaceae (buttercup family). Only two genera of the family, *Xanthorhiza* and *Clematis*, are shrubs or climbers, whereas the remaining 53 genera are almost entirely perennial, annual or biennial herbs (Supplementary Table 1). Ranunculaceae encompasses more than 2300 species worldwide, but most richly represented in the northern extratropical zone^30^. Most of the extant buttercup species dwell either in various forest types (e.g., evergreen/deciduous broad-leaved forests) or in open habitats (e.g., grasslands, meadows, or gravelly or rocky places) (Supplementary Table 1). Within temperate montane floras, the buttercup family ranks as the fourth or seventh in terms of species richness and is regarded as an important member of high-mountain ecosystems^31^. The buttercup fossil record highlights an age as old as the Lower Cretaceous for the family^32^. Thus, the family offers a remarkable opportunity for studying the evolution of ADHFs in the Cretaceous.

Based on a fossil-calibrated phylogeny of nearly all extant buttercup genera, we investigated the early tempo and mode of diversification of Ranunculaceae. We then evaluated: (1) whether the rise of forest-dwelling herbaceous angiosperm floras is simultaneous with the rise of angiosperm-dominated forests; (2) the timing of the rise of ADHFs in open vegetations; (3) the effect of the K-Pg mass extinction on ADHFs.

## Results and Discussion

### Phylogeny and divergence times

**Figure 1:**
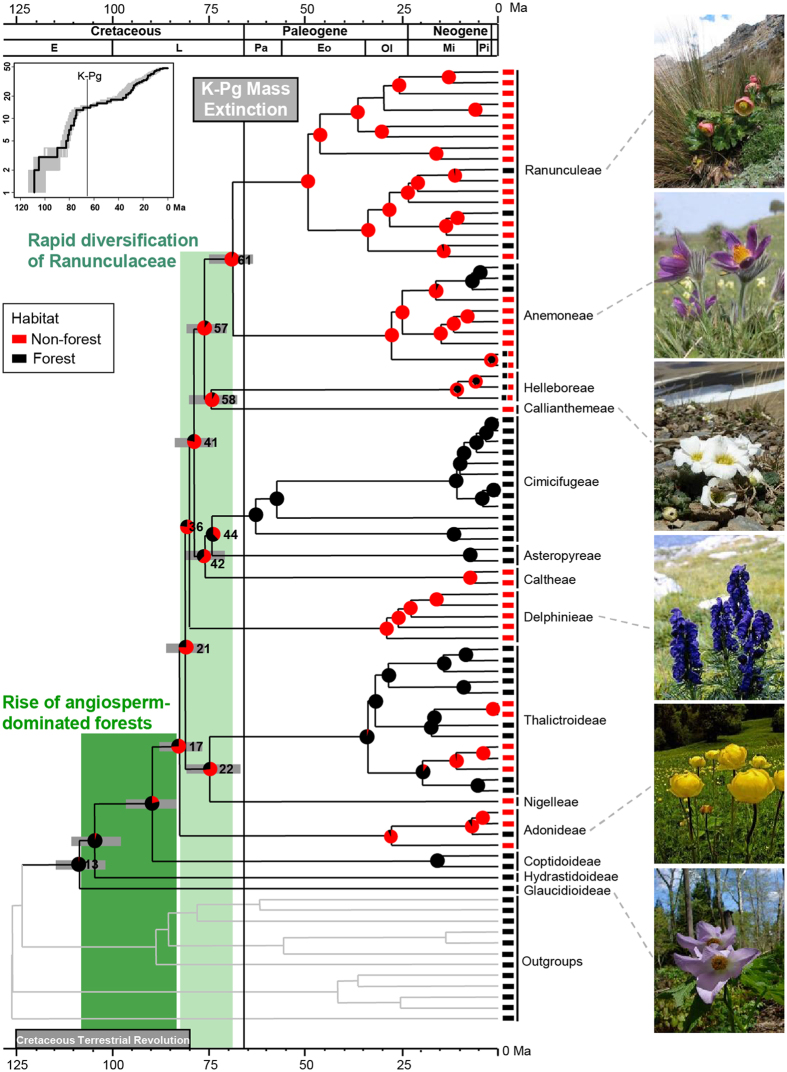
Phylogenetic chronogram and ancestral habitat reconstruction of extant Ranunculaceae. The timetree was generated by using all calibration points (analysis 1). Gray bars represent 95% highest posterior density of node age at subfamilial and tribal levels. Node numbers refer to Supplementary Fig. 2. Color-coded pie diagrams represent the probabilities of different states at each node under Bayesian inference. Our results indicate that the rapid diversification of Ranunculaceae (laurel-green shade) occurred after or at the end of the rise of angiosperm-dominated forests (green shade^20^). A standard LTT plot for the 108.79 Ma history of Ranunculaceae is present in the upper left, exhibiting a three-phase scenario in lineage accumulation before 48 Ma. Photographs by S.X.Y.

Fifty buttercup genera, representing 91% of the total extant generic diversity, were sequenced at six loci from plastid and nuclear genomes. Phylogenetic analyses recovered Glaucidioideae as basalmost in Ranunculaceae, followed by Hydrastidoideae and then Coptidoideae (Fig. 1 and Supplementary Figs 1 and 2), which are consistent with previous results^33^. Our results do not support a monophyletic Ranunculoideae, and instead suggest that Thalictroideae is nested in Ranunculoideae and sister to Nigelleae, although with no strong support. Most intergeneric relationships within Ranunculaceae are well resolved (Supplementary Fig. 1).

**Figure 2:**
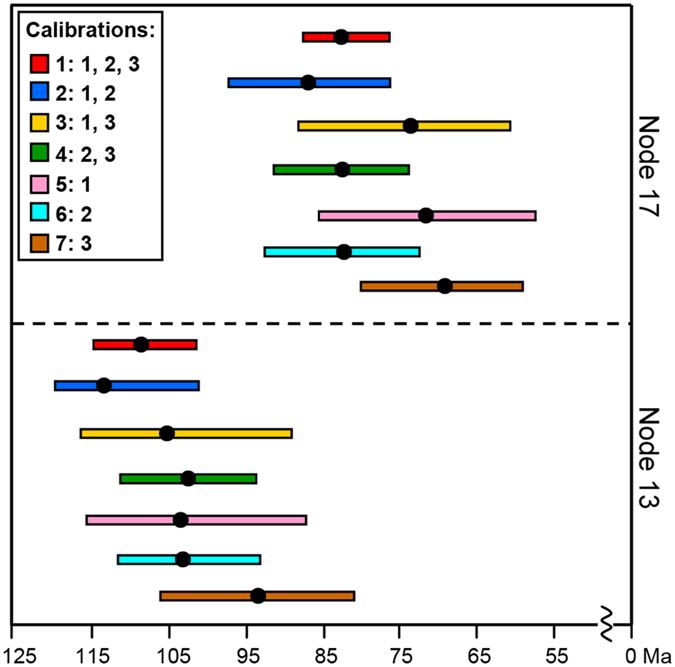
Summary of the crown age of Ranunculaceae (node 13) and the age of habitat shifts (node 17) under seven analytical scenarios. Dark dots represent mean ages. Horizontal bars represent 95% highest posterior density.

The seven calibration scenarios did not show significant differences for deep nodes except that the scenario with all three buttercup fossils produced narrower ranges of ages. For example, the confidence intervals for the crown age of the Ranunculaceae (node 13) and the age of habitat shifts (node 17) overlapped closely in all seven calibration scenarios, respectively (Fig. 2). The conservative estimation with all three calibrations is therefore reported (Supplementary Fig. 2 and Supplementary Table 2) and is the basis for the discussion. We dated the buttercup crown group to 108.79 Ma [101.57–114.57 Ma, 95% highest posterior density (HPD)], which is much older than many previous assumptions^34^,^35^. Previous studies had a poor taxon sampling for Ranunculaceae and no buttercup calibrations were used. Internal calibrations are deemed critical for obtaining accurate estimates^36^,^37^. A comparison of divergence time estimates for some clades or groups in Ranunculaceae is shown in Supplementary Table 3. Our results are supported by the cross-validation of one inferred node age against fossil age that was not used as calibration. The extinct *Eocaltha* was reported from the Campanian sediments of the Cerro del Pueblo Formation, Mexico. Based on seed characters, *Eocaltha* was postulated to be closely related to the extant *Caltha*^38^. The split between *Caltha* and its sister clade is here estimated at 76.18 (70.81–80.93) Ma, which corresponds well to the Campanian epoch (the Campanian ranges from 83.6 to 72.1 Ma^39^). Additionally, our fossil-calibrated time estimates suggest the split between *Aconitum* and *Gymnaconitum* at 26.02 (18.45–34.31) Ma, which falls in the time interval estimated earlier using a nuclear ITS rate calibration of 15–38 Ma^40^.

### Rise of angiosperm-dominated herbaceous floras in forests

Our estimate for the time of origin of crown group Ranunculaceae is at *c.* 108 Ma, the early mid-Cretaceous (Fig. 1), which corresponds to the beginning of the explosive radiation of angiosperms^5^,^6^,^7^,^41^ and the initial rise of angiosperm-dominated forests^20^. Our ancestral state reconstructions indicate that the buttercup origin was forest-dwelling and perennially herbaceous (Fig. 1 and Supplementary Fig. 3). After the origin of crown group, Ranunculaceae diversified into Hydrastidoideae and Coptidoideae, 104.85 (97.74–110.8) Ma and 89.9 (83.28–96.55) Ma, respectively. The two subfamilies both inhabit in angiosperm forests (Fig. 1). Our results therefore support that Ranunculaceae originated and diversified in forests during the mid-Cretaceous, *c.* 108–90 Ma, which coincides with the rise of angiosperm-dominated forests^20^,^27^,^42^,^43^.

The origin and early differentiation of Ranunculaceae in angiosperm forests also corresponds to that of other several forest-dwelling herbaceous angiosperm lineages. For example, Givnish *et al.* concluded that Orchidaceae, perhaps as the most diverse angiosperm family in forests, might have arisen *c.* 112 Ma and became differentiated at *c.* 90 Ma^44^. The origin of crown group Poaceae was estimated at *c.* 96 Ma and was postulated as forest-dwelling^45^. We therefore suggest that the rise of ADHFs in forests is almost synchronous with the rise of angiosperm-dominated forests, that is, herbaceous angiosperms in forest understories have diversified concurrent with woody angiosperms and hence in concert contributed to the KTR.

### Rise of angiosperm-dominated herbaceous floras in open vegetations

**Figure 3:**
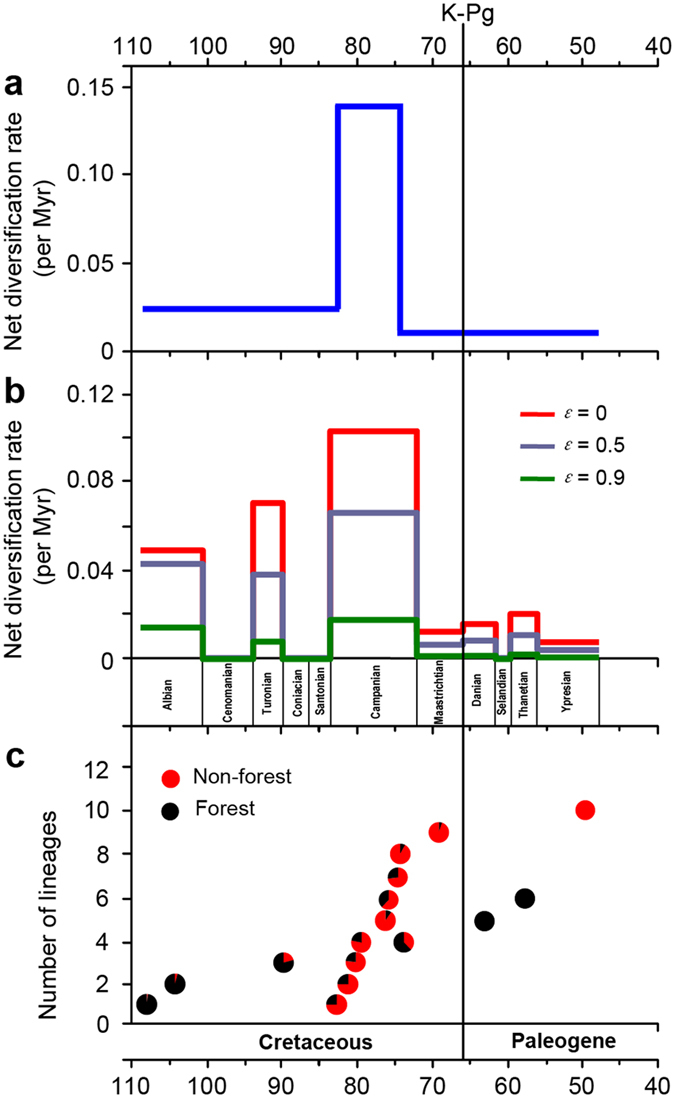
Early diversification dynamics of Ranunculaceae. (**a**) Net diversification rate estimates through time. (**b**) Average net diversification rates for each geological epoch. (**c**) Comparison between the accumulation of diversity in forest and non-forest buttercup lineages.

Taxon sampling in our data set is complete for lineages with extant buttercup descendants at 47.8 Ma (end of Ypresian) (Supplementary Table 4). The lineages-through-time (LTT) plots suggest a three-phase scenario in the accumulation of buttercup lineages until approximately 48 Ma, a dramatically increased rate at about 83 Ma and a slowdown around 74 Ma (Fig. 1, inset). TreePar analysis rejected the null hypothesis of the constant diversification rate of Ranunculaceae (χ^2^ = 6.66, *p* < 0.05). The model with two shifts (three-rate diversification model) fits the trees better. Ranunculaceae began to diversify at a rate of *r*_*1*_ = 0.025 species/Myr, followed by a shift at 82.8 Ma, increasing to *r*_*2*_ = 0.141 species/Myr, and another shift at 74.4 Ma, decreasing to *r*_*3*_ = 0.010 species/Myr (Fig. 3a). The stepwise plots of diversification rate through time indicate that net diversification rates are highest during the Campanian (Fig. 3b). Fossil data indicate that many angiosperms were herbaceous in the Campanian and Maastrichtian^46^. Dental complexity variations of multituberculate mammals increased sharply about 84 Ma, which led to an adaptive shift towards increased herbivory^47^. In this period, other herbivores, insect pollinators and dispersers also experienced a rapid diversification^48^.

**Figure 4:**
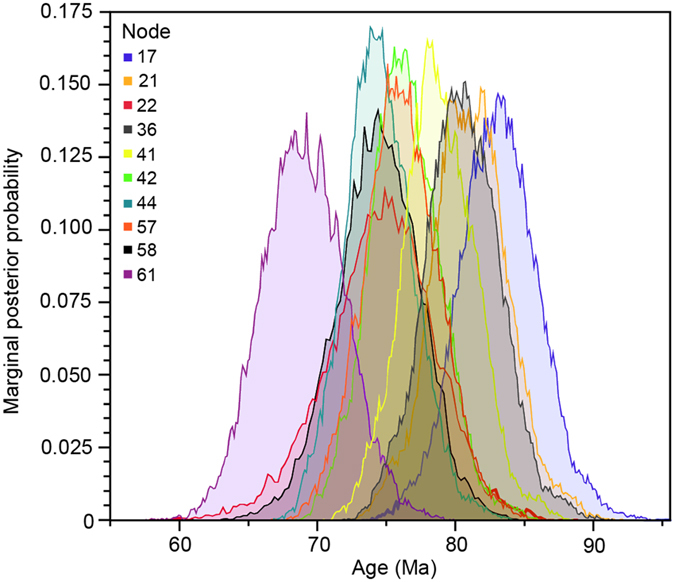
Posterior densities of estimated ages for the nodes of interest using all calibration points (analysis 1). Node numbers refer to Supplementary Fig. 2. Our results indicate that the full ranges of estimates obtained for these node ages are overlapping.

Our integration of time estimates and inference of ancestral habitat indicates that a habitat preference shift from forest to non-forest occurred at *c.* 83 Ma, the early Campanian (node 17). Subsequently, Ranunculaceae appears to have diversified rapidly into 11 herbaceous lineages (one subfamily and ten tribes), over a period of <14 million years (Myr), and perhaps in as little as 1 to 2 Myr (Figs 1 and 4, Supplementary Figs 2 and 3 and Supplementary Table 2). Among these 11 major lineages, seven are found in non-forests and three in forests (Fig. 1). Only *Helleborus* inhabit both habitats, however there is no one species entirely restricted to forests. We plotted the accumulation of lineages in forests and non-forests, and found that non-forest lineages accumulated faster than forest lineages from 82.8 Ma to 74.4 Ma (Fig. 3c). A phylogenetically unstructured comparison among subfamilies and tribes also indicates that the average net diversification rate for non-forest lineages is significantly greater than for forest-dwelling lineages (0.061 ± 0.025 species/Myr *vs.* 0.028 ± 0.028 species/Myr, *p* < 0.05). Thus, the elevated diversification of Ranunculaceae during the Campanian might mainly be ascribed to the rapid divergence of the non-forest lineages, possibly in response to a significantly global cooling (a drop of ~7 °C) during this period^49^,^50^.

We hypothesize that the burst in diversification of the non-forest Ranunculaceae corresponds to the rapid rise of ADHFs in open vegetations, mainly distributed in middle to high latitudes or altitudes. Compared with the rise of angiosperm-dominated forests (around 108 to 83 Ma)^20^, the rise of ADHFs in open vegetations was later, by the end of the KTR (Fig. 1). Angiosperms initially were woody species restricted to the lowlands of low latitudes, and later during the Cretaceous, spread towards higher latitudes or altitudes^51^,^52^,^53^. Fossil evidence has indicated that the ecological expansion of angiosperms was delayed at higher latitudes and altitudes^53^.

### Effect of the K-Pg mass extinction on angiosperm-dominated herbaceous floras

Our analyses suggest that all 14 buttercup lineages, which are stem branches leading to all extant subfamilies or tribes, are herbaceous and may have crossed the K-Pg boundary (Fig. 1 and Supplementary Fig. 3). There was no statistical support for a rate increase at or near the K-Pg boundary (Fig. 1, inset, and Fig. 3). We postulate that Ranunculaceae was relatively unaffected by the K-Pg mass extinction. Given that Ranunculaceae is a herbaceous angiosperm family mainly restricted to the northern extratropical zone^30^,^31^, we suggest that the K-Pg mass extinction had little effect on ADHFs, at least in the Northern Hemisphere, contradicting the viewpoint that terrestrial plant communities underwent a global deforestation at the K-Pg boundary^23^,^26^. In fact, the K-Pg mass extinction resulted from a rapid short-term global cooling^54^ could not kill all plant species, and instead selected against evergreen species^55^,^56^. Furthermore, the levels of extinction decreased towards high latitudes^56^. Multituberculate diversity increased continuously across the K-Pg boundary, which suggests that its food resources, mainly herbaceous angiosperms, might have experienced little change during the K-Pg extinction event^47^.

## Methods

### Molecular data sampling

We sampled 76 species from 50 of the 55 extant genera of Ranunculaceae, representing all five subfamilies and ten tribes of Ranunculoideae, and included twelve outgroups (Supplementary Table 5). We sequenced five plastid (*rbcL*, *matK*, *atpB*, *atpA*, and *ndhF*) and one nuclear (26S rDNA) gene regions accounting for a total of 8338 bp. When possible, data for each species were obtained from the same accession. Genomic DNA extraction, amplification, and sequence alignment were conducted as in Wang *et al.*^27^,^33^. The primers used in this study are listed in Supplementary Table 6.

### Phylogenetic analyses and divergence time estimates

The combined six-gene data set was analyzed using maximum likelihood (ML) and Bayesian inference (BI) methods. ML was performed in RAxML v7.0.4^57^ with 1000 replicates and the GTR + *Γ* model for each region. BI was performed in MrBayes v3.2.5^58^ by running four Markov Chain Monte Carlo (MCMC) chains with 50 million generations, and each DNA region assigned the best-fit model (*rbcL*, *atpA*, and 26S: GTR + I + *Γ*; *matK*: TVM + *Γ*; *atpB* and *ndhF*: TVM + I+ *Γ*; experimentally determined using jModeltest v2.1.4^59^).

Divergence times were co-estimated with phylogeny using a Bayesian relaxed-clock approach as implemented in BEAST v1.8.0^60^. Three buttercup fossils were selected as calibration points (see Supplementary Methods for more detail). (1) The split between Ranunculaceae and Berberidaceae was constrained to be 122.6 Ma based on the fossil *Leefructus mirus*^32^. (2) The split between *Actaea* and *Eranthis* was constrained to be 56.0 Ma based on the fossil *Paleoactaea nagelii*^61^. (3) A fossil achene of *Myosurus* was used to constrain its stem age of 23.03 Ma^62^. Following the recommendation of Parham *et al.*^63^, we enforced lognormal distributions for the three fossil calibration points as they better represent a hard minimum and soft maximum constraints. The offset (minimum age constraint) was set to be equal to the age of the fossil. The 95% upper bound of the distribution (soft maximum) was set by adjusting the standard deviation. Divergence times were also estimated using the three fossils in the following combinations: 1 and 2, 1 and 3, 2 and 3, 1, 2, and 3. In addition, a 125 Ma maximum age constraint was applied to the root in all seven dating analyses, which is the age of the oldest known tricolpate pollen^64^, with a normal distribution and a standard deviation of five.

All dating analyses were performed under an uncorrelated lognormal relaxed clock model of rate variation across branches, a Yule prior, and the GTR + I + *Γ* model for each gene partition separately. Parameters were estimated using four independent runs of 50 million generations each, with sampling every 2000 generations. Convergence was evaluated in Tracer v1.5. After a burn-in of 25%, the maximum clade credibility (MCC) tree with median branch lengths and a 95% HPD interval on nodes was compiled using TreeAnnotator v1.8.0.

### Ancestral state reconstructions

Most genera in Ranunculaceae are restricted in single habitat except that thirteen genera are found in various habitats (Supplementary Table 1). We first inferred the ancestral habitats of the thirteen genera based on the nine supplementary datasets (See Supplementary Methods). We then assigned the habitat state for each genus as either forest, which includes genera that dwell in forest understories, including rocky areas in forests. The alternative state, non-forest, includes variously open habitats (Supplementary Table 1). Habit for each genus was scored as woody, perennially herbaceous, or annually (including biennial) herbaceous. Habitat and habit data were obtained from the taxonomic literature, herbarium records, and field observations.

Ancestral state reconstructions were performed using a reversible-jump hyperprior (RJHP) MCMC approach in BayesTraits v2.0^65^ on 1000 subsampled posterior trees from the BEAST analysis with all three buttercup fossil constraints. This approach considers phylogenetic uncertainty^66^. The analysis was conducted using 100 million iterations, with sampling every 1000 iterations and a burn-in period of 10 million iterations, after adjusting the ratedev tuning parameter to ensure adequate mixing.

### Diversification analyses

Diversification analyses were performed on chronograms resulting from the BEAST analysis with all three buttercup fossil constraints. To visualize the temporal dynamics of diversification of Ranunculaceae, we first generated standard LTT plots in APE v3.1^67^, and then investigated the early diversification rates before 47.8 Ma (end of Ypresian). At this time, the truncated timetrees for Ranunculaceae included all lineages that gave rise to living descendants (See Supplementary Methods and Supplementary Table 4). TreePar^28^ was used to identify the locations of temporal shifts in diversification rates of Ranunculaceae. TreePar analyses were run with a grid setting of 0.1 million years under the pure birth model. The significance of rate shifts was determined using the likelihood-ratio test^28^. We also calculated the average net diversification rates for each geological epoch in Geiger v1.99-3^29^ with three relative extinction rates (*ε* = 0/0.5/0.9).

Additionally, a phylogenetically unstructured analysis was used to compare differences between forest-dwelling and open-vegetation clades (at the subfamilial and tribal levels, all of which occurred prior to 47.8 Ma) in net diversification rate *D* = (ln *S*)/*t*, where *S* is number of extant species in a clade and *t* is its stem age^68^.

## Additional Information

**How to cite this article**: Wang, W. *et al.* The rise of angiosperm-dominated herbaceous floras: Insights from Ranunculaceae. *Sci. Rep.* **6**, 27259; doi: 10.1038/srep27259 (2016).

## Supplementary Material

Supplementary Information
