# Supplementary material for: The rise of angiosperm-dominated herbaceous floras: Insights from Ranunculaceae
Source: Sci Rep. 2016 Jun 2;6:27259. doi: 10.1038/srep27259 (PMC4890112; doi:10.1038/srep27259)
Supplement: Supplementary Information [file srep27259-s1.pdf]

Additional information

The rise of angiosperm-dominated herbaceous floras: Insights from Ranunculaceae

Wei Wang[1], Li Lin[1], Xiao-Guo Xiang[1], Rosa del C. Ortiz[2], Yang Liu[3], Kun-Li Xiang[1], Sheng-Xiang Yu[1], Yao-Wu Xing[4] & Zhi-Duan Chen[1]

[1] State Key Laboratory of Systematic and Evolutionary Botany, Institute of Botany, Chinese Academy of Sciences, Beijing 100093, China. [2] Missouri Botanical Garden, P.O. Box 299, St. Louis, Missouri 63166-0299, USA. [3] Department of Ecology and Evolutionary Biology, University of Connecticut, Storrs, CT 06269-3043, USA. [4] Field Museum of Natural History, 1400 S Lake Shore Drive, Chicago, IL 60605, USA.

Correspondence and requests for materials should be addressed to W.W. (email: wangwei1127@ibcas.ac.cn) or to Z.-D.C. (email: zhiduan@ibcas.ac.cn)

Running head: The rise of angiosperm-dominated herbaceous floras

Supplementary Figure 1 Phylogram obtained from ML analysis of the six-marker data set.

Supplementary Figure 2 Time-calibrated tree of extant Ranunculaceae using all calibration points (analysis 1).

Supplementary Figure 3 Reconstruction of ancestral habits of extant Ranunculaceae.

Supplementary Figure 4 Ancestral habitat reconstructions for thirteen genera of Ranunculaceae with two or more habitat types.

Supplementary Table 1 Genera of Ranunculaceae indicating the number of recognized and sampled species, habits, and habitats.

Supplementary Table 2 Crown group ages of all nodes in the phylogeny.

Supplementary Table 3 Comparison of divergence time estimates (Ma) for some clades or groups in Ranunculaceae.

Supplementary Table 4 Evaluation of candidate lineages that may have originated prior to 47.8 Ma.

Supplementary Table 5 Taxa, voucher and GenBank accession numbers for the sequences used in this study.

Supplementary Table 6 Primers used for amplification and sequencing in this study.

Supplementary Table 7 Additional accession numbers (not listed in Supplementary Table 5) for supplementary molecular dating analyses and ancestral habitat reconstructions.

Supplementary Methods

Legends for Supplementary Figures 1–4

Supplementary Figure1 | Phylogram obtained from ML analysis of the six-marker data set. Numbers above and below branches are bootstrap percentages (> 50%) and Bayesian posterior probabilities (> 0.50), respectively. (-) denotes lack of support for the clade in the Bayesian inference method. Subfamilies and tribes are based on Wang *et al.*[2].

Supplementary Figure 2 | Time-calibrated tree of extant Ranunculaceae using all calibration points (analysis 1). Nodes are at mean divergence times and gray bars represent 95% highest posterior density of node age. Node numbers refer to Supplementary Table 2. The numbers in circles show the locations of fossil calibration points.

Supplementary Figure 3 | Reconstruction of ancestral habits of extant Ranunculaceae. Node numbers refer to Supplementary Figure 2.

Supplementary Figure 4 | Ancestral habitat reconstructions for thirteen genera of Ranunculaceae with two or more habitat types. (A) *Aquileiga*, (B) *Thalictrum*, (C) Adonideae, (D) Anemoneae, (E) *Clematis*, (F) *Caltha*, (G) Delphinideae, (H) *Helleborus*, (I) *Ranunculus*.

Supplementary Table 1 | Genera of Ranunculaceae indicating the number of recognized and sampled species, habits, and habitats. Data are obtained from several sources (Tamura[1]; Electronic databases: www.efloras.org; personal observation in fields and herbarium specimens). Subfamilial and tribal classification is based on Wang *et al.*[2]. The genera are mainly based on Tamura[1], however the newly delimited or resurrected genera after Tamura[1] are based on Wang *et al.*[3] and references therein.

Subfamiliy	Tribe	Genus	No. species	No. spp. sampled	Habit	Habitat
Glaucidioideae		*Glaucidium*	1	1	Perennial	Alpine or mountain woodlands
Hydrastidoideae		*Hydrastis*	1	1	Perennial	Deciduous forests
Coptidoideae		*Coptis*	15	1	Perennial	Deciduous forests (*C. trifoliolata* grows in alpine meadows, which is distributed in more derived clade).
		Xanthorhiza	1	1	Shrubby	Woods and thickets
Thalictroideae		*Aquilegia*	*c.* 80	2	Perennial	Meadows, forests, or rocky or gravelly places. Based on our inference of habitat evolution in the *Aquileiga*, the most recent common ancestor (MRCA) of the extant *Aquilegia* inhabited in open vegetaions (Supplementary Fig. 4A).
		Dichocarpum	19	3	Perennial	Forests
		Enemion	6	1	Perennial	Deciduous forest
		Isopyrum	2	1	Perennial	Deciduous or maxed forests
		Leptopyrum	1	1	Annual	Open grasslands, wastelands or farms
		Paraquilegia	5	1	Perennial	Rocky places in the alpine zone
		Paropyrum	2	1	Perennial	Rocky or grassy places, or crevices of rocks
		Semiaquilegia	1	1	Perennial	Forests
		Thalictrum	*c.* 200	2	Perennial	Rocky, sandy, grassy, or boggy places, grasslands, bushes or forests. Based on our inference of habitat evolution in the *Thalictrum*, the MRCA of the extant *Thalictrum* inhabited in forests (Supplementary Fig. 4B).
		Urophysa	2	1	Perennial	Rocky places or crevices of rocks in forests
Ranunculoideae	Adonideae	*Adonis*	*c.* 26	1	Perennial or annual	Grasslands, rocky places or forests. Based on our inference of habitat evolution in the Adonideae, the MRCA of the extant *Adonis* inhabited in open vegetations (Supplementary Fig. 4C).
		Calathodes	4	1	Perennial	Mountain forests, open slopes. Based on our inference of habitat evolution in the Adonideae, the MRCA of the extant *Calathodes* inhabited in forests (Supplementary Fig. 4C).

	Trollius (incl. *Megaleranthis*)	32	2	Perennial	Meadows, herbaceous or gravelly fields, or deciduous forests. Based on our inference of habitat evolution in the Adonideae, the MRCA of the extant *Trollius* inhabited in meadows (Supplementary Fig. 4C).
Anemonea	*Anemoclema*	1	1	Perennial	Alpine grasslands
	Anemone (incl. *Pulsatilla, Knowltonia, Oreithales, Barneoudia*)	*c.* 150	4	Perennial, rarely shrubby	Grasslands, thickets, tundras, snowbeds, sandy or stony places, or forests. Based on our inference of habitat evolution in the Anemoneae, the MRCA of the extant *Anemone* inhabited in meadows (Supplementary Fig. 4D).
	Clematis (incl. *Archiclematis, Navavelia*)	*c.* 250	3	Scandent shrubby	Forests, bushes, or grasslands. Based on our inference of habitat evolution in the *Clematis*, the MRCA of the extant *Clematis* inhabited in forests (Supplementary Fig. 4E).
	Hepatica	*c.* 70	2	Perennial	Our phylogenetic analyses indicate that *Hepatica* and the group with $x = 7$ in *Anemone* form a clade, which is sister to the remaining Anemoneae. Here, we defined the clade as a broad *Hepatica*. Based on our inference of habitat evolution in the Anemoneae, the MRCA of the extant broad *Hepatica* inhabited in grasslands or forests (Supplementary Fig. 4C).
	Metanemone	1	0	Perennial	Alpine grasslands
Asteropyreae	*Asteropyrum*	2	2	Perennial	Forests
Callianthemeae	*Callianthemum*	14	1	Perennial	Meadows, rocky, stony or boggy places
Caltheae	*Caltha*	12	2	Perennial	Grasslands, meadows, marshes, water, or forests. Based on our inference of habitat evolution, the MRCA of the extant *Caltha* inhabited in open vegetations (Supplementary Fig. 4F).
Cimicifugeae	*Actaea* (incl. *Cimicifuga, Souliea*)	28	9	Perennial	Deciduous forests
	Anemonopsis	1	1	Perennial	Montane deciduous forests
	Beesia	2	1	Perennial	Forests
	Eranthis	8	1	Perennial	Deciduous of mixed forests, or thickets, sometimes in open places
Delphinieae	*Aconitum*	*c.* 300	1	Perennial	Forests, grasslands, bushes, or grassy or rocky places. Based on our inference of habitat evolution in the Delphinieae, the MRCA of the extant *Aconitum* inhabited in open vegetaions (Supplementary Fig. 4G).
	Delphinium (incl. *Consolida*)	*c.* 370	2	Perennial or annual	Forests, grasslnds, bushes, or grassy or rocky places, boulders, screes, barrens, semidesert. Based on our inference of habitat evolution in the Delphinieae, the MRCA of the extant *Delphinium* inhabited in open vegetaions (Supplementary Fig. 4G).
	Gymnaconitum	1	1	Annual or Biennial	Grassy or sandy places
	Staphisagria	3	1	Annual or Biennial	Rocky places (adaped to disturbed habitats)

Helleboreae	*Helleborus*	*c.* 20	3	Perennial	Forests, scrubs, or grassy or rocky places. Based on our inference of habitat evolution in the *Helleborus*, the MRCA of the extant *Helleborus* inhabited in forests or open vegetations (Supplementary Fig. 4H).
Nigelleae	*Garidella*	2	0	Annual	Grasslands, wastelands or farms
	Komaroffia	2	0	Annual	Steppes, weed-infestd places or cultivated fields
	Nigella	*c.* 20	1	Annual	Grasslands, grassy or rocky places, or fallow or cultivated fields
Ranunculeae	*Arcteranthis*	1	0	Perennial	Damp slopes and rocky crevices near snow
	Beckwithia	1	1	Perennial	Slopes in sagebrush or pinyon-juniper woodland
	Callianthemoides	1	1	Perennial	Near the lower limit of permanent snow
	Ceratocephala	4	1	Annual	Open, grassy places
	Coptidium	2	1	Perennial	Bogs or tundras
	Cyrtorhyncha	1	1	Perennial	Rocky places, cliffs or ravines
	Ficaria	5	1	Perennial	Meadows, forest margins, fields
	Halerpestes	*c.* 10	1	Perennial	Solonchaks or solonetzic soils or bogs, meadows, lake shores or seashores
	Hamadryas	6	1	Perennial	Gravelly, rocky or sparsely grassy places
	Krapfia	*c.* 8	1	Perennial	Grasslands
	Kumlienia	1	1	Perennial	Forests
	Laccopetalum	1	1	Perennial	Rock-clefts, gravelly places, sometimes marshes near the snow line
	Myosurus	15	1	Annual	Grassy or stony places, disturbed or cultivated fields
	Oxygraphis	4	1	Perennial	Meadows or tunders
	Paroxygraphis	1	0	Perennial	Wet places
	Peltocalathos	1	1	Perennial	Damp gullies, streamsides and the base of wet cliffs
	Ranunculus (incl. *Aphanostemma*)	*c.* 600	3	Perennial or annual	Grasslands, forests, tundras, or waters. Based on our inference of habitat evolution in the *Ranunculus*, the MRCA of the extant Ranunculus inhabited in grasslands (Supplementary Fig. 4I).
	Trautvetteria	3	1	Perennial	Damp grassy places

Supplementary Table 2 | Crown group ages of all nodes in the phylogeny. Node numbers refer to Supplementary Figure 2.

Node	Age (Ma)	95% highest posterior density
1	126.4	125.58–127.26
2	126.22	125.35–127.08
3	41.47	32.01–54.16
4	25.36	17–34.67
5	36.45	25.32–47.44
6	123.72	123.27–124.3
7	88.94	71.13–100.39
8	85.69	69.2–97.53
9	55.79	38.65–73.14
10	13.68	8.05–21.75
11	78.22	62.05–90.8
12	62	46.9–75.65
13	108.79	101.57–114.75
14	104.85	97.74–110.8
15	89.9	83.28–96.55
16	16.23	8.51–5.96
17	82.78	76.73–87.93
18	27.68	18.13–41.98
19	6.82	4.35–10.44
20	4.01	2.21–6.59
21	81.32	76.08–86.06
22	74.93	66.89–81.06
23	33.81	27.62–41.24
24	19.69	12.56–28.08
25	5.33	2.24–9.34
26	10.9	6.66–16.99
27	3.89	1.5–7.55
28	31.95	26.27–39.36
29	17.38	13.52–24.14
30	16.59	12.83–23.36
31	1.2	0.4–2.53
32	28.55	20.78–34.92
33	9.04	5.58–13.2
34	14.23	9.45–19.96
35	8.51	4.6–13.16
36	80.23	
37	28.95	20.89–39.13
38	26.02	18.45–34.31
39	22.64	15.73–30.36
40	16.18	10.29–22.91
41	79.01	73.81–83.97

42	76.18	70.81–80.93
43	7.39	4.1–11.94
44	74.42	
45	7.38	3.64–11.93
46	63.04	60.02–66.71
47	11.43	5.9–19.37
48	57.55	56.41–59.21
49	10.75	8.25–13.5
50	4.02	2.21–6.67
51	0.38	0.02–0.97
52	9.93	7.51–12.52
53	8.86	6.44–11.45
54	5.67	3.92–7.9
55	3.19	1.86–4.87
56	1.23	0.48–2.21
57	76.38	70.37–80.88
58	74.52	67.66–80.2
59	10.46	5.81–16.85
60	6.02	2.86–10.66
61	68.95	63.44–75.08
62	27.63	22.55–32.62
63	2.06	0.8–3.9
64	24.93	20.36–31.9
65	14.79	10.84–19.4
66	11.64	8.28–15.92
67	8.16	4.64–12.1
68	16.22	12.08–20.93
69	6.76	4.56–9.58
70	5.04	2.73–7.56
71	49.4	42.72–54.92
72	33.89	25.84–43.03
73	14.34	5.54–28.45
74	28.24	20.99–36.07
75	13.54	6.92–20.85
76	10.49	5.46–18.06
77	23.23	17.32–30.58
78	20.96	14.56–28.49
79	11.23	4.53–18.93
80	46.3	40.1–51.92
81	16.15	5.42–31.55
82	36.45	31.28–42.16
83	30.4	25.32–35.46
84	29.65	24.37–36.33
85	25.68	20.33–31.88

86	6.2	2.18–12.72
87	13.05	8.47–18.03

Supplementary Table 3 | Comparison of divergence time estimates (Ma) for some clades or groups in Ranunculaceae.

Clade/group	This study	Prior study	Comments
Thalictroideae$_{crown}$	33.81 (27.62–41.24)	27.61 (26.59–28.56)[4]	Our estimate for the Thalictroideae crown group age is slightly older than that of Bastida *et al.*[4]. The latter used the split age of *Ranunculus* and *Xanthorhiza* (58.0 ± 2.5 Ma) reported by Wikström *et al.*[5], as well as the oldest endocarp fossil of Menispermaceae, as calibration points. However, in Wikström *et al.*[5], only five genera in Ranunculaceae were included.
Aquilegia/*Urophysa* split	17.38 (13.52–24.14)	10.18 (9.21–11.14)[4]	Our age for the Thalictroideae crown group is much older than that of Bastida *et al.*[4]. See above comments for the Thalictroideae crown age.
Delphinieae$_{crown}$	28.95 (20.89–39.13)	32.3 (23.0–41.8)[6]	Our age for the Delphinieae crown group is highly consistent with that of Jabbour & Renner[6]. These two ages also overlapped closely with that obtained from nuclear ITS rate calibration[7].
Ranunculeae$_{crown}$	49.2 (42.72–54.92)	38.36 (28.64–47.04)[8]<br>38.85 (32.07–40.04)[9]	Our age for the Ranunculeae crown gourp is older than that of Emadzade & Hoerandl[8] and Wang *et al.*[9]. These two studies constrained the split of Ranunculeae and Anemoneae at 46.6 Ma, which was estimated by Anderson *et al.*[10]. However, Anderson *et al.*[10] did not use any buttercup fossil as calibratin point. In this study, we used three buttercup fossils as calibration points and dated the split of Ranunculeae and Anemoneae at 68.95 Ma (Supplementary Table2).
Anemone/*Clematis* split	24.93 (20.36–31.9)	25.99 (9.19–43.77)[11]	The split age of *Anemone* and *Clematis* estimated in this study is highly consistent with that obtained by Xie *et al.*[11].

Supplementary Table 4 | Evaluation of candidate lineages that may have originated prior to 47.8 Ma. Asterisks denote candidate lineages that were included in supplementary molecular dating analyses.

Taxon	Comments
Glaucidium	*Glaucidium palmatum* is the only living species in Glaucidioideae
Hydrastis	*Hydrastis canadensis*is the only living species in Hydrastidoideae
Coptidoideae	Our taxon sampling includes both extant genera in the subfamily. The age of this subfamily is 16.23 Ma based on our analyses.
Thalictroideae	Our taxon sampling includes all ten extant genera in the subfamily. The age of this subfamily is 33.81 Ma based on our analyses.
Nigelleae*	Nigelleae includes three extant genera (*Garidella*, *Komaroffia*, and *Nigella*). We included all three genera of Nigelleae in supplementary analyses, and recovered a date of 42.7 Ma for the crown group of the tribe.
Delphinieae	Our taxon sampling includes all four extant genera in the tribe. The age of this tribe is 28.95 Ma based on our analyses, which is slightly less than the result of Jabbour & Renner[6] (32.3Ma).
Caltheae*	Caltheae only consists of one genus, *Caltha*, which includes 12 species. We included all extant species of the genus in supplementary analyses, and recovered a date of 37.7 Ma for the crown group of the genus.
Asteropyreae	Our taxon sampling includes both extant species in the tribe. The age of this tribe is 7.38 Ma based on our analyses.
Beesia	*Beesia* includes two extant species. Our taxon sampling indexes the split between the genus and *Anemonopsis* at 11.43 Ma.
Anemonopsis	*Anemonopsis* only includes one extant species.
Eranthis	*Eranthis* includes eight extant species in Cimicifugeae and is distributed in Eurasia. We sampled seven extant species of the genus in supplementary analyses, and recovered that the crown group age of the genus is < 47 Ma.
Actaea	The new delimited *Actaea* of Compton *et al.*[12] includes three genera of Tamura[2], *Actaea*, *Cimicifuga*, and *Souliea*. Our taxon sampling includes all three genera, and indexes that they differentiated at 10.75 Ma (Supplementary Table 2).
Callianthemeae*	Callianthemeae only consists of one genus, *Callianthemum*, which includes ca. 14 species distributed in Eurasia. We included three species (two species from Europe and one species from East Asia) of the genus in supplementary analyses, and recovered a date of 3.19 Ma for the crown group of the genus.
Helleboreae*	Helleboreae only consists of one genus, *Helleborus*, which includes ca. 20 species distributed in Eurasia. We included 20 accessions, representing 17 species of the genus in supplementary analyses, and dated the basal split in *Helleborus* at 17.21 Ma.
Anemoneae	The basal split within this taxon (*Hepatica* vs. remaining Anemoneae) is indexed by our taxon sampling and is < 48 Ma.
Metanemone	*Metanemone* only has one extant species in Anemoninae of Anemoneae. The description of the genus was based on only one specimen, and no other specimens than the type have been collected so far. Based on Tamura[2], the Anemoninae includes *Anemone*, *Barneoudia*, *Hepatica*, *Knowltonia*, *Metanemone*, *Oreithales*, and *Pulsatilla*. Molecular phylogenetic studies suggest that *Barneoudia*, *Knowltonia*, *Oreithales*, and *Pulsatilla* were reduced to *Anemone* [1,13]. Our taxon sampling indicates the split between the broad *Anemone* and Clematidinae at 24.93 Ma
Paroxygraphis	*Paroxygraphis* only has one extant species in Ranunculeae. To date, the genus has not been sampled in any molecular study. Wang *et al.*[9] sampled 18 of the 19 genera in Ranunculeae and found that the tribe was composed of two major clades (I and II). Based on the fruit and beak characters, *Paroxygraphis* can be placed in clade II. Wang *et al.*[9] dated the basal split in clade II at 20-23 Ma. Our taxon sampling indicates the basal split in clade II at 33.89 Ma (Supplementary Table 2).
Beckwithia	*Beckwithia* only includes one extant species.
Cyrtorhyncha	*Cyrtorhyncha* only includes one extant species.
Peltocalathos	*Peltocalathos* only includes one extant species.
Callianthemoides	*Callianthemoides* only includes one extant species.
Hamadryas	Our taxon sampling indexes the split between the genus and *Callianthemoides* at 10.49 Ma.
Halerpestes	The stem age of the genus is 23.23 Ma base on our analyses.
Oxygraphis	The stem age of the genus is 20.96 Ma base on our analyses.

Arcteranthis	*Arcteranthis* only has one extant species in Ranunculeae. Wang *et al.*[9] dated the split between the genus and *Trautvetteria* at 5–7 Ma.
Kumlienia	*Kumlienia* only includes one extant species.
Trautvetteria	Our taxon sampling indexes the split between the genus and *Trautvetteria* at 11.23 Ma.
Coptidium	Our taxon sampling indexes the split between the genus and *Ficaria* at 16.15 Ma.
Ficaria	Our taxon sampling indexes the split between the genus and *Coptidium* at 16.15 Ma.
Ceratocephala	Our taxon sampling indexes the split between the genus and *Myosurus* at 36.45 Ma.
Myosurus	Our taxon sampling indexes the split between the genus and *Ceratocephala* at 36.45 Ma.
Laccopetalum	*Laccopetalum* only includes one extant species.
Krapfia	Our taxon sampling indexes the split between the genus and *Laccopetalum* at 6.2 Ma.
Ranunculus	Our taxon sampling indexes the split between the genus and its sister group at 29.65 Ma.
Adonideae	Our taxon sampling includes all three extant genera in the tribe. The age of this tribe is < 48 Ma based on our analyses.

Supplementary Table 5 | Taxa, voucher and GenBank accession numbers for the sequences used in this study.

Taxon	Vouchers	GenBank accession numbers					
**Ranunculaceae**		*rbcL*	*matK*	*atpB*	*atpA*	*ndhF*	26S rDNA
Aconitum racemulosum Franch.	Wang Wei 081 (PE)	AY954488	FJ626484	KU662814	KU662742	KU662883	AY954473
Actaea asiatica Hara	Chen et al. 960060 (PE)	FJ626575	FJ626485	KU662815	KU662743	KU662884	FJ626436
Actaea erythrocarp Fischer	Zhou Y CBS053 (PE)	KU662943	KU662872	KU662816	KU662744	KU662885	KU662723
Adonis amurensis Regel & Radde	Chen Z-D 003 (PE)[1]; S. Hoot 933 (UWM)[2]	AY954487[1]	FJ626486[1]	EU053870[2]	KU662745[1]	KU662886[1]	AY954472[1]
Anemoclema glaucifolium (Franch.) W. T. Wang	Wang Wei YN070 (PE)	FJ626576	FJ626487	KU662817	KU662746	KU662887	FJ626437
Anemone hupehensis Lem.	Wang Wei 002 (PE)	FJ626577	FJ626488	KU662818	KU662747	KU662888	FJ626438
Anemonopsis macrophylla Sieb. & Zucc.	A. Reznicek 9977 (MICH)	FJ626578	FJ626489	EU053874	KU662748	KU662889	AF131289
Aquilegia ecalcarata Maxim.	Wang Wei 117 (PE)	AY954495	EF437127	KU662819	KU662749	KU662890	AY954481
Aquilegia oxysepala Trautv. & Kir.	Chen Z-D 001 (PE)	EF437140	EF437128	KU662820	KU662750	KU662891	FJ626439
Asteropyrum cavaleriei (Lévl. et Vant.) Drumm. & Hutch.	Wang & Chen, unpubl.[1]; Wang Wei 110 (PE)[2]	AF079453[1]	FJ626490[2]	KU662821[2]	KU662751[2]	KU662892[2]	AY954466[2]
Asteropyrum peltatum (Franchet) Drumm. & Hutch.	Chen J-W *s.n.* (PE)	KU662944	KU662873	KU662822	KU662752	KU662893	KU662724
Batrachium bungei (Steud.) L. Liou	Wang Wei YN092 (PE)	FJ626579	FJ626491	KU662823	KU662753	KU662894	FJ626440
Beckwithia andersonii (A. Gray) Jeps.	J.T. Johansson *s.n.* (GB)		AY954238				
Beesia calthifolia (Maxim.) Ulbr.	Wang and Chen (unpubl.)[1]; Chen 200010661(PE)[2]	AF079452[1]	FJ626492[2]	KU662824[2]	KU662754[2]	KU662895[2]	AY954468[2]
Calathodes oxycarpa Sprague	Wang 940823 (PE)	KU662945	HQ440174	KU662825	KU662755	KU662896	KU662725
Callianthemoides semiverticillatus (Philippi) Tamura	C. Lehnebach *s.n.* (VALD)		AY954236				
Callianthemum taipaicum W. T. Wang	Wang Wei SX004 (PE)	FJ626580	FJ626493	KU662826	KU662756	KU662897	FJ626441
Caltha palustris var. *membranacea* Turcz.	Chen Z-D2072 (PE)	FJ626581	FJ626494	KU662827	KU662757	KU662898	FJ626442
Caltha leptosepala DC.	Chen Z-D CL111 (PE)	KU662946	KU662874	KU662828	KU662758	KU662899	KU662726
Ceratocephala orthoceras DC.	ZDY427 (XJBI)[1]; E. Hörandl 3837 (WU)[2]	KU662947[1]	AY954230[2]	KU662830[1]	KU662760[1]	KU662900[1]	KU662727[1]
Cimicifuga acerina (Sieb. et Zucc.) Tanaka	Chen et al. 981298 (PE)[1]; Song et al. (2001)[2]	KU662948[1]	AF353578[2]	KU662831[1]	KU662761[1]	KU662901[1]	KU662728[1]
Cimicifuga brachycarpa Hsiao	Wang Wei 057 (PE)	KU662949	KU662875	KU662832	KU662762	KU662902	KU662729

Cimicifuga dahurica (Turcz.) Maxim.	Gu Jing 09 (PE)	KU662950	KU662876	KU662833	KU662763	KU662903	KU662730
Cimicifuga foetida L.	Chen Z-D 961202 (PE)	KU662951	KU662877	KU662834	KU662764	KU662904	KU662731
Cimicifuga racemosa (L.) Nutt.	R. Phillippe et al. 42571 (PE)	KU662952	KU662878	KU662835	KU662765	KU662905	KU662732
Cimicifuga simplex Wormsk.	A. Reznicek 9238 (MICH)[1]; Li et al. (unpubl)[2]; Chen and Xu 2076 (PE)[3]	EU053908[1]	AB044754[2]	EU053878[1]	KU662766[3]	KU662906[3]	KU662733[3]
Clematis ganpiniana (Lévl. & Vant.) Tamura	Wang Wei 119 (PE)	AY954491	FJ626495	KU662836	KU662767	KU662907	AY954476
Clematis henryi Oliv.	Chen Z-D JGS035 (PE)	KU662953	KU662879	KU662837	KU662768	KU662908	KU662734
Consolida ajacis (L.) Schur	Wang Wei Seed2 (PE)	FJ626582	FJ626496	KU662838	KU662770	KU662909	FJ626443
Coptidium pallasii (Schlecht.) Tzvelev	R. Elven et al. SUP02-175 (O)		AY954233				
Coptis chinensis Franch.	Chen et al. 960105 (PE)	AY954497	DQ478614	KU662839	KU662771	KU662910	AY954482
Cyrtorhyncha ranunculina Nutt.	S. Nunn 1775 (RM)		GU257981				
Delphinium bonvalotii Franch.	Wang Wei 030 (PE)	FJ626583	FJ626497	KU662840	KU662772	KU662911	FJ626444
Dichocarpum sutchuenense W. T. Wang & Hsiao	Wang Wei 069 (PE)	AY954493	EF437130	KU662841	KU662773	KU662912	AY954479
Dichocarpum dalzielii (Drumm. et. Hutch.) W. T. Wang et Hsiao	Wang Wei 111 (PE)	EF437141	EF437129	KU662842	KU662774	KU662913	
Dichocarpum fargesii W. T. Wang & Hsiao	Guo CC QL-152 (PE)	KU662954	KU662880	KU662843	KU662775	KU662914	KU662735
Enemion raddeanum Regel	Chen Z-D 2090 (PE)	AY954494	EF437131	KU662845	KU662777	KU662915	AY954478
Eranthis stellata Maxim.	Chen Z-D 2040 (PE)	AY954484	FJ626498	KU662847	KU662779	KU662916	AY954467
Ficaria verna ssp. *verna* Huds.	S. Hoot 002 (UWM)[1]; J.T. Johansson *s.n.*[2]; M. Wayda *s.n.* (PE)[3]	EU053919[1]	AY954232[2]	EU053890[1]	KU662780[3]	KU662917[3]	KU662736[3]
Glaucidium palmatum Sieb. & Zucc.	Hoot 924 (UWM)[1]; Adachi et al., unpubl.[2]; Zhou J 001 (PE)[3]	AF093723[1]	AB069850[2]	KU662848[3]	KU662781[3]	KU662918[3]	AF3892674[1]
Gymnaconitum gymnandrum (Maxim.) Wei Wang & Z.D. Chen	Dickoré 9111 (MSB)	JF331677	JF331792				
Hamadryas magellanica Lam. (*delfinii* Phil. for *matK*)	E. Pisano et al. 8251 (CONC)[1]; P. Schönswetter AR08-20 (WU)[2]	EU053914[1]	GU257982[2]	EU053884[1]			
Halerpestes ruthenica (Jacq.) Ovcz.	ZDY 339 (XJBI)[1]; Sukachev 1519 (LE)[2]	KU662955[1]	FM242773[2]	KU662850[1]	KU662783[1]	KU662919[1]	KU662737[1]
Helleborus sp.	Wang Wei 090409 (PE)	KU662956	KU662881	KU662851	KU662784	KU662920	KU662738
Helleborus thibetanus Franch.	Wang Wei SX 032 (PE)	AY954485	FJ626500	KU662852		KU662921	AY954470
Helleborus orientalis Lam.	S. Hoot 9224 (UWM)[1]; K. Heo 208 (KWNU)[2]	EU053915[1]	AY515247[2]	EU053885[1]			
Hepatica henryi (Oliv.) Steward	Chen 200010660 (PE)	FJ626584	FJ626501	KU662853	KU662785	KU662922	FJ626445

Hepatica nobilis Mill.	Chen 2007 (PE)[1]; Stuessy et al. 17626 (WU)[2]	KU662957[1]	DQ994672[2]	KU662854[1]	KU662786[1]	KU662923[1]	KU662739[1]
Hydrastis canadensis L.	Zaczi 2883 (MICH)[1]; Adachi et al., unpubl.[2]; Chen Z-D 2002016 (PE)[3]; YDK 92-9 (TEX)[4]	AF093725[1]	AB069849[2]	AF093382[1]	KU662787[3]	AY145146[4]	AF389268[1]
Isopyrum manshuricum Kom.	Wang Wei LN004 (PE)	EF437143	EF437133	KU662855	KU662788	KU662924	FJ626446
Krapfia ranunculina DC. (*clypeata* DC. for *matK*)	A. Hofreiter C25 (MSB)[1]; I. Sanchez-Vega et al. 11173 (F)[2]	EU053916[1]	DQ490058[2]	EU053886[1]			
Knowltonia vesicatoria (L.f.) Sims	Shiraishi *s.n.*[1]; Chen Z-D 200980 (PE)[2]		AB110533[1]	KU662857[2]	KU662790[2]	KU662925[2]	
Kumlienia hystricula (A. Gray) Greene	L. Grant 286 (ZT)		GU257983				
Laccopetalum giganteum (Wedd.) Ulbr.	J. Pera *s.n.* (HUT)[1]; Cano et al. 15196 (USM)[2]	EU053931[1]	DQ400695[2]	EU053887[1]			
Leptopyrum fumarioides (L.) Reichb.	Man Y-G T101 (PE)	EF437145	EF437135	KU662858	KU662791	KU662926	
Megaleranthis saniculiflia Ohwi	Kim et al., 2009[1]; Yoo No. 67 (KWNU)[2]; Ro K-E (355)[3]	NC_012615[1]	AY515243[2]	NC_012615[1]	NC_012615[1]	NC_012615[1]	AF131285[3]
Myosurus minimus L.	28.4.1962 Hans Luther (UPS)[1]; Hoot 98-2 (UWM)[2]	DQ099441[1]	FJ626502[2]	EU053888[2]	KU662794[2]	KU662927[2]	FJ626447[2]
Naravelia zeylanica (L.) DC.	Wang Wei YN126 (PE)	FJ626585	FJ626503	KU662859	KU662795	KU662928	KU662740
Nigella damascena L.	Wang Wei Seed1 (PE)	FJ626586	FJ626504	EU053889	KU662796	KU662929	FJ626448
Oreithales integrifolia (DC.) Schltdl.	M. Weigend et al. 7503 (K)	KU662958	KU662882	KU662860	KU662797	KU662930	KU662741
Oxygraphis glacialis (Fisch.) Bunge.	Wang Wei SX006 (PE)	FJ626587	FJ626505	KU662861	KU662798	KU662931	FJ626449
Paraquilegia microphylla (Royle) Drumm. & Hutch.	Li C-Y 001 (PE)	EF437146	EF437136	KU662862	KU662800	KU662932	FJ626450
Paropyrum anemonoides (Kar. & Kir.) Ulbr.	Wundish U. 177 (PE)	EF437142	EF437132	KU662863	KU662801		
Peltocalathos baurii (MacOwan) Tamura	L. Mucina 030103/22 (WU)		AY954235				
Pulsatilla cernua (Thunb.) Bercht. & Opiz.	Chen Z-D 2048 (PE)	AY954492	AB110531	KU662864	KU662802	KU662933	AY954477
Ranunculus cantoniensis DC.	Wang Wei 031 (PE)	AY954489	FJ626506	KU662865	KU662803	KU662934	AY954474
Ranunculus macranthus Scheele	RCH1184 (TEX)	DQ069502	DQ069586	DQ069346	DQ069340	NC_008796	
Semiaquilegia adoxoides (DC.) Makino	Shao Q 001 (PE)	EF437147	EF437137	KU662866	KU662805	KU662935	FJ626451
Souliea vaginata (Maxim.) Franch.	Wang Wei SX015 (PE)	FJ626588	FJ626507	KU662867	KU662806	KU662936	FJ626452
Staphisagria requienii (DC.) Spach	Erben *s.n.* (coll. date 6.6.1991) (M)	JF332021	JF331806				
Thalictrum javanicum Bl.	Wang Wei 067 (PE)	AY954496	DQ478615	KU662868	KU662807	KU662937	AY954480
Thalictrum robustrum Maxim.	Wang Wei 038 (PE)	EF437148	EF437138	KU662869	KU662808	KU662938	
Trautvetteria carolinensis (Walt.) Vail.	Hoot 9218 (UWM)	FJ626589	FJ626508	EU053896	KU662810	KU662939	U52630

Trollius laxus Salisb.	Chen ZD CL108 (PE)	AY954486	FJ626509	KU662870	KU662811	KU662940	AY954471
Urophysa henryi (Oliv.) Ulbr.	Wang Wei 096 (PE)	EF437149	EF437139	KU662871	KU662812	KU662941	FJ626453
Xanthorhiza simplicissima Marshall	QiuY-L 91030 (UCN)[1]; Adachi et al., unpubl.[2]; Kim et al., 2004[3]	L12669[1]	AB069848[2]	AF093394[1]	KU662813[1]	KU662942[1]	AF389270[3]
**Berberidaceae**							
Caulophyllum robustum Maxim.	Xiang et al., 2000[1]; Adachi et al., unpubl.[2]; Chen et al. 960601 (PE)[3]; YDK 92-5 (TEX)[4]	AF190441[1]	AB069832[2]	KU662829[3]	KU662759[3]	AY145149[4]	FJ626455[3]
Diphylleia cymosa Michx.	J. Wen 997 (A)[1]; Chen Z-D 2002019 (PE)[2]	L75866[1]	DQ478620[2]	KU662844[2]	KU662776[2]	AY145154[1]	FJ626456[2]
Epimedium koreanum Nakai	Y-D Kim 92-8 (TEX) [1]; Adachi et al., unpubl.[2]; Zhou Y *s.n.* (PE)[3]	L75869[1]	AB069837[2]	KU662846[3]	KU662778[3]	AY145163[1]	FJ626459[3]
Gymnospermium microrrhynchum Takht.	Chen Z-D 2047 (PE)[1]; Adachi et al., unpubl.[2]; YDK 92-13 (TEX)[3]	EF173671[1]	AB069833[2]	KU662849[1]	KU662782[1]	AY145161[3]	FJ626460[1]
Jeffersonia diphylla (L.) Pers.	Y-D Kim 91-1 (TEX)[1]; Adachi et al., unpubl.[2]; Chen 2002010 (PE)[2]; Ro et al., 1997[4]	L75867[1]	AB069836[2]	KU662856[3]	KU662789[3]	AY145152[1]	U52604[4]
Mahonia bealei (Fort.) Carr.	Qiu 74 (NCU)[1]; Chen et al. 960046 (PE)[2]	L12657[1]	DQ478617[2]	AF197611[2]	KU662792[2]	JN051733[2]	FJ626461[2]
Nandina domestica Thunb.	Y-D. Kim 94-1 (TEX)[1]; Adachi et al., unpubl.[2]; Hoot 922 (UWM)[3]; M. J. Moore 310 (FLAS)[4]	L75843[1]	AB069830[2]	FJ026397[3]	NC_008336[4]	AY145148[1]	AF389241[3]
**Menispermaceae**							
Cocculus trilobus (Thunb.) DC.	Hong Y-P H310 (PE)	JN051678	DQ478611	JN051873	KU662769	JN051704	FJ626466
Menispermum canadense L.	Soltis & Soltis 2526 (WS)[1]; Naczi (2937)[2]; Ortiz et al. 235 (MO)[3]	AF190437[1]	GU266604[1]	AF093384[2]	KU662793[3]	EF624311[3]	AF389257[1]
Parabaena sagittata Miers	Hong Y-P H346 (PE)	FJ626597	EF143854	HQ260849	KU662799	JN051714	FJ626470
Tinomiscium petiolare Hook. f. & Thoms.	Hong Y-P H142 (PE)	EF173675	DQ478612	HQ260863	KU662809	JN051732	FJ626474
**Lardizabalaceae**							
Sargentodoxa cuneata (Oliv.) Rehd. & Wils.	Hong Y-P 99238 (PE)[1]; Qin *s.n.* (PE)[2]; Pan 93001 (NCU)[3]	FJ626605[1]	FJ626515[1]	AF093396[2]	KU662804[1]	JN051734[1]	DQ008620[3]

Supplementary Table 6 | Primers used for amplification and sequencing in this study.

Locus	Name	Sequence (5’-3’)	References
rbcL	1F	ATGTCACCACAAACAGAAACT	Chen *et al.*[14]
	991R	CGGTACCAGCGTGAATATGAT	Chen *et al.*[14]
	1494R	GATTGGGCCGAGTTAATTAC	Chen *et al.*[14]
matK	AF2	CTTTCAGGARTACATTTATGC	Wang *et al.*[15]
	8R2	ACGWGCCAAAGTTCTAGCAC	Wang *et al.*[15]
	mF2	AAACAATCTTMTCATTTACG	Wang *et al.*[15]
	mR2	AARGGATCCTTGAACAMCCA	Wang *et al.*[15]
atpB	2F	TATGAGAATCAATCCTACTACTTCT	Hoot *et al.*[16]
	1494R	TCAGTACACAAAGATTTAAGGTCA	Hoot *et al.*[16]
	611F	CGTACTCGTGAAGGAAATGA	Hoot *et al.*[16]
	1186R	TGTCCTGAAGTTCTTTGTAACGTTG	Hoot *et al.*[16]
atpA	418F	GTCCGACAACGAGTTTT(TC)CAACAAGC	Schuettpelz *et al.*[17]
	535F	ACAGCAGTAGCCAC(AG)GATAC	This study
	1079R	TCC(TC)GGATA(AT)GCTTC(AG)CGACC	This study
	46F	GTATAGGTTC(AG)A(AG)TCCTATTGGACG	Schuettpelz *et al.*[17]
ndhF	1F	ATGGAACAKACATATSAATATGCGTGG	Olmstead & Sweere[18]
	980F	GCMTATTCTACAATGTCTC	Wang *et al.*[19]
	1318R	CGAAACATATAAAATGCRGTTAATCC	Olmstead & Sweere[18]
26S rDNA	F1	TAAGCATATNAMTAAGCGGAG	Wang *et al.*[20]
	R4	ATARTTCACCATMTTTCGGG	Wang *et al.*[20]
	R5	TGCTACTACCACCAAGATC	Wang *et al.*[20]

Supplementary Table 7 | Additional accession numbers (not listed in Supplementary Table 5) for supplementary molecular dating analyses and ancestral habitat reconstructions.

Supplementary analyses	Taxon			
**1. Caltheae and Callianthemeae**		ITS	*atpB-rbcL*	*trnL-trnF*
	Caltha appendiculata	AY365385	AY365402	AY365366
	Caltha dionaeifolia	AY365389	AY365403	AY365367
	Caltha introloba	AY365387	AY365404	AY365368
	Caltha natans	AY365398	AY365407	AY365371
	Caltha novae-zelandiae	AY365388	AY365408	AY365372
	Caltha obtusa	AY365386	AY365409	AY365373
	Caltha sagittata	AY365391	AY365412	AY365378
	Caltha scaposa	AY365396	AY365414	AY365379
	*Caltha palustris (*Japan)	AY365397	AY365410	AY365374
	Caltha palustris (USA)	AY365382	AY365415	AY365376
	Caltha palustris (France)	AY365392	AY365411	AY365375
	Caltha palustris (Altai)	KF233850	KF233830	KF233870
	Caltha palustris var. *membranacea*	AY515398		AY515398
	Caltha palustris var. *umbrosa*	KF233852	KF233832	KF233872
	Caltha palustris var. *barthei*	KF233851	KF233831	KF233871
	Caltha palustris var. *sibirica*	KF233853	KF233833	KF233873
	Caltha rubriflora	KF233854	KF233834	KF233874
	Caltha sinogracilis	KF233857	KF233837	KF233877
	Caltha leptosepala ssp. *howellii*	AY365395	AY365405	AY365369
	Caltha leptosepala ssp. *leptosepala*	AY365394	AY365406	AY365370
	Callianthemum anemonoides	AY365390	AY365400	AY365364
	Callianthemum coriandrifolium	AY365393	AY365401	AY365365
	Callianthemum taipaicum	Unpubl.	Unpubl.	FJ626539
	Anemonopsis macrophylla	Z98275		AJ222984
	Trollius ledebouri	AY365383	AY365417	AY365381
**2. *Helleborus***		ITS	*trnL-F*	*matK*
	Helleborus argutifolius	AJ347920	AJ413279	AJ414317
	Helleborus atrorubens	AJ347888	AJ413280	AJ414318
	Helleborus croaticus	AJ347889	AJ413281	AJ414319
	Helleborus cyclophyllus	AJ347890	AJ413282	AJ414320
	Helleborus dumetorum	AJ347891	AJ413284	AJ414321
	Helleborus foetidus	AJ347892	AJ413283	AJ414322
	Helleborus lividus	AJ347893	AJ413285	AJ414323
	Helleborus multifidus ssp. *bocconei*	AJ347894	AJ413286	AJ414324
	Helleborus multifidus ssp. *hercegovinus*	AJ347895	AJ413287	AJ414325
	Helleborus multifidus ssp. *istriacus*	AJ347896	AJ413288	AJ414326
	Helleborus multifidus ssp. *multifidus*	AJ347897	AJ413289	AJ414327
	Helleborus niger	AJ347898	AJ413290	AJ414328

	Helleborus odorus	AJ347899	AJ413292	AJ414329
	Helleborus orientalis	AJ347901	AJ413293	AJ414331
	Helleborus purpurascens	AJ347902	AJ413294	AJ414332
	Helleborus thibetanus	AJ347904	AJ413296	AJ414334
	Helleborus torquatus	AJ347905	AJ413297	AJ414335
	Helleborus vesicarius	AJ347907	AJ413299	AJ414337
	Helleborus viridis ssp. *viridis*	AJ347908	AJ413300	AJ414338
	Helleborus viridis ssp. *occidentali*	AJ347909	AJ413301	AJ414339
	Anemoclema glaucifolium	Unpubl.	FJ626535	FJ626487
	Callianthemum taipaicum		FJ626539	FJ626493
	Caltha palustris var. *membranacea*	AY515398	FJ626540	FJ626494
**3. Nigelleae**		ITS		
	Nigella arvensis ssp. *aristata*	EU699439		
	Nigella arvensis ssp. *arvensis*	EU699440		
	Nigella arvensis ssp. *brevifolia*	EU699441		
	Nigella arvensis ssp. *glauca*	EU699442		
	Nigella carpatha	EU699443		
	Nigella ciliaris	EU699445		
	Nigella damascena	EU699446		
	Nigella degenii ssp. *barbro*	EU699447		
	Nigella degenii ssp. *jenny*	EU699449		
	Nigella doerfleri	EU699450		
	Nigella elata	EU699451		
	Nigella fumariifolia	EU699454		
	Nigella gallica	EU699461		
	Nigella icarica	EU699456		
	Nigella orientalis	EU699457		
	Nigella oxypetala	EU699459		
	Nigella papillosa	EU699462		
	Nigella sativa	EU699463		
	Nigella segetalis	EU699465		
	Nigella stellaris	EU699467		
	Garidella nigellastrum	EU699471		
	Garidella unguicularis	EU699472		
	Komaroffia diversifolia[1]	EU699474		
	Komaroffia diversifolia[2]	EU699475		
	Adonis vernalis	AF454936		
	Caltha natans	AY365398		
	Caltha palustris	AY365382		
	Thalictrum robustum	EF437125		
	Dichocarpum dalzielii	EF437115		
**4. *Eranthis***		ITS	*trnL-F*	
	Eranthis byunsanensis	JF505772	JF505898	
	Eranthis hyemalis	Z98273	AJ222982	

	Eranthis lobulata	Unpubl.	Unpubl.	
	Eranthis longistipitata	Unpubl.	Unpubl.	
	Eranthis pinnatifida	AJ496615	AJ496611	
	Eranthis pungdoensis	JF505797	JF505923	
	Eranthis stellata	FJ597992	FJ626543	
	Actaea rubra	Z98278	AJ222987	
	Actaea spicata	Z98279	AJ222988	
	Souliea vaginata	KF233847	KF233867	
	Cimicifuga foetida	Z98287	AJ222995	
	Cimicifuga americana	Z98280	AJ222989	
	Cimicifuga japonica	Z98291	AJ222999	
	Anemonopsis macrophylla	Z98275	AJ222984	
	Beesia calthifolia	AJ496613	AJ496612	
	Asteropyrum cavaleriei	KF233846	KF233866	
	Caltha natans	AY365398	AY365371	
	Trollius ledebouri	AY365383	AY365381	

**5. Adonideae**		ITS	*trnL-F*	*matK*
	Adonis amurensis	AB361619	FJ626534	FJ626486
	Adonis annua	AY148280	AH012590	JN895412
	Adonis brevistyla	KF233849	KF233869	
	Adonis multiflora	AB361621	AB361607	
	Adonis pseudoamurensis	AF454935		
	Adonis ramosa	AB361611	AB361597	
	Adonis shikokuensis	AB361623	AB361609	
	Adonis vernalis	AF454936	AJ413302	AJ414340
	Calathodes oxycarpa	HQ440197	HQ440186	HQ440174
	Calathodes palmata	HQ440198	HQ440175	HQ440175
	Calathodes polycarpa	Unpubl.	Unpubl.	Unpubl.
	Megaleranthis saniculifolia	AY515399	HQ440188	AY515243
	Trollius acaulis	Unpubl.	Unpubl.	Unpubl.
	Trollius altaicus		AH012580	AY515233
	Trollius asiaticus	AY148267	AH012571	HQ440176
	Trollius chinensis	AY148268	AH012572	AY515235
	Trollius dschungaricus	HQ440199	HQ440189	HQ440177
	Trollius europaeus	HQ440200	HQ440190	HQ440178
	Trollius farreri	HQ440201	HQ440191	HQ440179
	Trollius japonicus	AY148278	AH012587	AY515240
	Trollius laxus	AY148266	AH012578	FJ626509
	Trollius ledebouri	AY148271	AH012579	AY515237
	Trollius macropetalus	HQ440202	HQ440192	HQ440180
	Trollius membranostylis	AY148272	AH012581	HQ440181
	Trollius micranthus	Unpubl.	Unpubl.	Unpubl.
	Trollius pumilus	AY148273	AH012582	
	Trollius ranunculinus	AY148277	AH012584	

	Trollius ranunculoides	HQ440203	HQ440193	HQ440182	
	Trollius riederianus	AY148279	AH012588	AY515241	
	Trollius sibiricus	HQ440204	HQ440194	HQ440183	
	Trollius vaginatus	HQ440205	HQ440195	HQ440184	
	Trollius yunnanensis	HQ440206	HQ440196	HQ440185	
	Trollius yunnanensis var. *auemonifolius*	Unpubl.	Unpubl.	Unpubl.	
	Trollius yunnanensis var. *peltatus*	Unpubl.	Unpubl.	Unpubl.	
	Aconitum racemulosum	AY150233	FJ626533	FJ626484	
	Anemone hupehensis	HQ440207	FJ626536	FJ626488	
	Beesia calthifolia	AJ496613	AJ496612	FJ626492	
	Caltha palustris var. *membranacea*	AY515398	FJ626540	FJ626494	
	Eranthis stellata	FJ597992	FJ626543	FJ626498	
	Helleborus thibetanus	AJ347904	AJ413296	FJ626500	
**6. Anemoneae**		ITS	*atpB-rbcL*	*matK*	*rbcL*
	Anemone amurensis	EF139270			
	Anemone antucensis	AY056049	AF311735		
	Anemone apennina	JF422883			
	Anemone berlandieri	FJ639876	FJ639841		
	Anemone blanda	AY055402	AY055422		
	Anemone caffra	AY055399	AY055420		
	Anemone canadensis	AY055387.	AY055408		EU053902
	Anemone capensis	JF810688	JF810698		
	Anemone caroliniana	AY055403	AY055423		
	Anemone coronaria	FJ639877	AF386086		
	Anemone crassifolia	AY055398	AY055419		
	Anemone cylindrica	JF422889			JX848515
	Anemone decapetala	FJ639878	FJ639843		
	Anemone demissa	AY055392	AY055413		
	Anemone drummondii	AY055404	AY055424		
	Anemone edwardsiana	FJ639880	FJ639845		
	Anemone fanninii	JF810685	JF810695		
	Anemone flaccida	AY055391	AY055412	AB110530	
	Anemone helleborifolia	FJ639881	FJ639846		
	Anemone hortensis	FJ639882	FJ639847		
	Anemone hupehensis	AY055397	AY055418	FJ626488	FJ626577
	Anemone keiskeana	AY055390	AY055411		
	Anemone koraiensis	FJ597985			
	Anemone lithophila	FJ639883	FJ639848		
	Anemone mexicana	FJ639884	FJ639849		
	Anemone narcissiflora	AY055393	AY055414		
	Anemone nemorosa	AM267278	AF386091	JN895407	JN891147
	Anemone obtusiloba	AY055394	AY055415		
	Anemone palmata		AF386087		
	Anemone parviflora	FJ639887	FJ639851	JN966091	JN965244

Anemone pavonina	FJ639888	AF386092		
Anemone pendulisepala	EF139262			
Anemone quinquefolia	GU257978	JF810696		
Anemone raddeana	EF139249			
Anemone ranunculoides	FJ639889	FJ639853		
Anemone reflexa	EF139275			
Anemone richardsonii	AY055388	AY055409	JN966093	JN965246
Anemone rigida	FJ639890	FJ639854		
Anemone rivularis	AY055396	AY055417		
Anemone rupicola	FJ639892	FJ639856		
Anemone sellowii	FJ639893	FJ639857		
Anemone somaliensis	JF810687	JF810697		
Anemone stolonifera	EF139243			
Anemone sylvestris	FJ639894	FJ639858		
Anemone tenuicaulis	AY055389	AY055410		
Anemone tenuifolia	JF810689	JF810699		AM235090
Anemone tetrasepala	FJ639895	FJ639859		
Anemone thomsonii	JF810690	JF810700		
Anemone triternata	FJ639896	FJ639860		
Anemone trullifolia	AY055395	AY055416		
Anemone tuberosa	FJ639897	FJ639861		
Anemone virginiana	DQ006033	AF386088	HQ593170	HQ589955
Anemone vitifolia	FJ639899	FJ639863		
Barneoudia balliana	FJ639900	FJ639864		
Barneoudia chilensis	FJ639901	FJ639865		
Barneoudia major	FJ639902	FJ639866		
Hepatica acutiloba	AM267285	AM267300	DQ994677	HQ589952
Hepatica americana	AY055386	AY055407	AF542590	EU053901
Hepatica falconeri	EF012263		DQ994675	
Hepatica henryi	AM267290	AM267297	DQ994674	FJ626584
Hepatica insularis	AM267288	AM267298	DQ994673	
Hepatica maxima	AM267282	AM267295	DQ994671	
Hepatica nobilis var. *asiatica*	AM267289	AM267296	DQ994672	
Hepatica nobilis var. *japonica*	AB120214	AM267292	AB110532	
Hepatica nobilis var. *nobilis*	AM267284	AM267294	DQ994668	
Hepatica nobilis var. *pubescens*	AM267287	AM267293	DQ994667	
Hepatica transsilvanica	AM267283	AM267299	DQ994670	
Knowltonia anemonoides	JF810691	JF810701		
Knowltonia bracteata	JF810692	JF810702		
Knowltonia capensis	JF810703	JF810693		
Knowltonia cordata	JF810694		AB110533	
Knowltonia vesicatoria	AY055401	AY055421		
Oreithales integrifolia	Unpubl.	Unpubl.	Unpubl.	Unpubl.
Pulsatilla albana	JF422890			

	Pulsatilla cernua	Unpubl.	Unpubl.	AB110531	AY954492		
	Pulsatilla chinensis	GU732650	GU732569				
	Pulsatilla dahurica	GU732648	GU732567				
	Pulsatilla halleri	FJ639908	FJ639872				
	Pulsatilla koreana	GU732647	GU732566				
	Pulsatilla montana	AM267281					
	Pulsatilla occidentalis	AY055400	AY055426		EU053903		
	Pulsatilla patens	AM267280					
	Pulsatilla rubra	JF422891					
	Pulsatilla turczaninovii	GU732649	GU732568				
	Pulsatilla violacea	JF422893					
	Anemoclema glaucifolium	Unpubl.	Unpubl.	FJ626487	FJ626576		
	Clematis afoliata	AJ347911	AB115443	AB110512			
	Clematis alternata			AB110509			
	Clematis angustifolia			AB110534			
	Clematis fasciculiflora			AB110521			
	Clematis fusca			AB110535			
	Clematis ganpiniana	Unpubl.		AY954491	FJ626495		
	Clematis henryi	Unpubl.		Unpubl.	Unpubl.		
	Clematis lasiantha	GU732601	GU732520	AB110518			
	Clematis orientalis			AB110515			
	Clematis potaninii	GU732619	GU732538	AB110517			
	Clematis sibirica	GU732630	GU732549				
	Clematis tashiroi			AB110511			
	Clematis williamsii			AB110500			
	Naravelia zeylanica	Unpubl.	Unpubl.	FJ626585	FJ626503		
	Trautvetteria caroliniensis	FJ639909	FJ639875	FJ626589	FJ626508		
	Halerpestes ruthenica	FM242837	Unpubl.	Unpubl.	FM242773		
	Ranunculus cantoniensis	Unpubl.	Unpubl.	AY954489	FJ626506		
	Ficaria verna	AY680192	FJ639874	EU053919	AY954232		
**7. *Clematis***		ITS	*atpB-rbcL*	*psbA-trnQ*	*rpoB-trnC*	*trnK-matK*	*rbcL–accD*
	Clematis acerifolia	GU732572	GU732491	GU732653	GU732734		
	Clematis aethusifolia	GU732573	GU732492	GU732654	GU732735		
	Clematis afoliata	AJ347911	AB115443	AB117586	AB116890	AB110512	AB116982
	Clematis akebioides	GU732574	GU732493	GU732655	GU732736		
	Clematis alpina	GU732576	GU732495	GU732657	GU732738		
	Clematis alternata	AB120190	AB115440	AB117582	AB116887	AB110509	AB116979
	Clematis angustifolia	AB120199	AB115449	AB117591	AB116896	AB110534	AB116988
	Clematis apiifolia	GU732577	GU732496	AB117572	GU732739	AB110499	AB116969
	Clematis armandii	GU732578	GU732497	GU732659	GU732740		
	Clematis brachiata	GU732579	GU732498	GU732660	GU732741	JF270693	
	Clematis brachyura	AB120204	AB115454	AB117596	AB116901	AB110522	AB116993
	Clematis brevicaudata	GU732583	GU732502	GU732664	GU732745		
	Clematis chinensis	GU732584	GU732503	GU732665	GU732746		

Clematis chrysocoma	GU732586	GU732505	GU732667	GU732748		
Clematis confusa	GU732588	GU732507	GU732669	GU732750		
Clematis crassifolia	AB120194	AB115444	AB117585	AB116891	AB110513	AB116983
Clematis crispa	GU732589	GU732508	GU732670	GU732751		
Clematis delavayi	AB120202	AB115452	AB117594	AB116899	AB110520	AB116991
Clematis drummondii	GU732591	GU732510	GU732672	GU732753		
Clematis eichleri	AB120209	AB115459	AB117601	AB116906	AB110527	AB116998
Clematis erectisepala	GU732592	GU732511	GU732673	GU732754		
Clematis fasciculiflora	AB120203	AB115453	AB116900	AB116900	AB110521	AB116992
Clematis finetiana	GU732593	GU732512	GU732674	GU732755		
Clematis florida	AB120186	AB115436	AB117578	AB116883	AB110505	AB116975
Clematis fusca	AB120179	AB115429	AB117571	AB116876	AB110535	AB116968
Clematis gentianoides	AB120210	AB115460	AB117602		AB110528	AB116999
Clematis glycinoides	GU732595	GU732514	GU732676	GU732757		
Clematis heracleifolia	GU732596	GU732515	GU732677	GU732758		
Clematis hexapetala	GU732597	GU732516	GU732678	GU732759		
Clematis integrifolia	GU732599	GU732518	GU732680	GU732761		
Clematis intricata	JN809683					
Clematis japonica	AB120187	AB115437	AB117579	AB116884	AB110506	AB116976
Clematis lasiandra	AB120185	AB115435	AB117577	AB116884	AB110504	AB116974
Clematis lasiantha	AB120200	AB115450	AB117592	AB116897	AB110518	AB116989
Clematis leschenaultiana	GU732603	GU732522	GU732684	GU732765		
Clematis ligusticifolia	AB120201	AB115451	AB117593	AB116898	AB110519	AB116990
Clematis linearifolia	GU732606	GU732525	GU732687	GU732768		
Clematis loureiroana	GU732607	GU732526	GU732688	GU732769		
Clematis mandshurica	GU732608	GU732527	GU732689	GU732770		
Clematis meyeniana	GU732609	GU732528	GU732690	GU732771		
Clematis microphylla	HM116960					
Clematis montana	GU732610	GU732529	GU732691	GU732772		
Clematis nobilis	AB120206	AB115456	AB117598	AB116903	AB110524	AB116995
Clematis ochotensis	AB120182	AB115432	AB117574	AB116879	AB110501	AB116971
Clematis orientalis	AB120196	AB115446	AB116893	GU732774	AB110515	AB116985
Clematis paniculata	DQ499135					
Clematis patens	AB120184	AB115434	AB117576	AB116881	AB110503	AB116973
Clematis peterae	GU732614	GU732533	GU732695	GU732776		
Clematis pierotii	AB120191	AB115441	AB117583	AB116888	AB110510	AB116980
Clematis pinnata	GU732616	GU732535	GU732697	GU732778		
Clematis pitcheri	GU732617	GU732536	GU732698	GU732779		
Clematis pogonandra	GU732618	GU732537	GU732699	GU732780		
Clematis potaninii	AB120198	AB115448	AB117590	AB116895	AB110517	AB116987
Clematis pubescens	GU732620	GU732539	GU732701	GU732782		
Clematis ranunculoides	GU732621	GU732540	GU732702	GU732783		
Clematis recta	GU732623	GU732542	GU732704	GU732785		
Clematis rehderiana	GU732624	GU732543	GU732705	GU732786		

Clematis reticulata	GU732625	GU732544	GU732706	GU732787		
Clematis rutoides	GU732626	GU732545	GU732707	GU732788		
Clematis serratifolia	AB120205	AB115455	AB117597	AB116902	AB110523	AB116994
Clematis siamensis	GU732629	GU732548	GU732710	GU732791		
Clematis sibirica	GU732630	GU732549	GU732711	GU732792		
Clematis stans	AB115438	AB115438	AB116885	AB116885	AB110507	AB116977
Clematis strigillosa	GU732631	GU732550	GU732712	GU732793		
Clematis taiwaniana	GU732632	GU732551	GU732713	GU732794		
Clematis tangutica	AB120195	AB115445	AB117587	AB116892	AB110514	AB116984
Clematis tashiroi	AB120192	AB115442	AB117584	AB116889	AB110511	AB116981
Clematis terniflora	AB120183	AB115433	AB117575	AB116880	AB110502	AB116972
Clematis texensis	AB120197	AB115447	AB117589	AB116894	AB110516	AB116986
Clematis tibetana	GU732635	GU732554	GU732716	GU732797		
Clematis uncinata	GU732637	GU732556	GU732718	GU732799	AB110508	AB116978
Clematis villosa	AB120211	AB115461	AB117603	AB116908	AB110529	AB117000
Clematis virginiana	GU732638	GU732557	GU732719	GU732800	HQ593242	
Clematis vitalba	AB120207	AB115457	AB117599	AB116904	AB110525	AB116996
Clematis williamsii	AB120181	AB115431	AB117573	AB116878	AB110500	AB116970
Clematis ganpiniana	Unpubl.				Unpubl.	
Clematis henryi	Unpubl.				Unpubl.	
Clematis javana	DQ499136					
Clematis macropetala	JN809684					
Naravelia laurifolia	GU732646	GU732565	AB117600	AB116905	AB110526	AB116997
Naravelia zeylanica	Unpubl.	Unpubl.			Unpubl.	
Pulsatilla chinensis	GU732650	GU732569	GU732731	GU732812		
Pulsatilla dahurica	GU732648	GU732567	GU732729	GU732810		
Pulsatilla koreana	GU732647	GU732566	GU732728	GU732809		
Pulsatilla turczaninovii	GU732649	GU732568	GU732730	GU732811		
Hepatica nobilis var. *japonica*	AB120214	AB115464		AB116911	AB110532	AB117003
Pulsatilla cernua	Unpubl.	Unpubl.	AB117605	AB116910	AB110531	AB117002
Anemone rivularis	GU732571	GU732490	GU732652	GU732733		
Knowltonia sp.	AB120215	AB115465	AB117606	AB116912		AB117004
Anemone flaccida	AY055391	AY055412	AB117604	AB116909		AB117001
Ranunculus macranthus		NC_008796	NC_008796	NC_008796	NC_008796	NC_008796
Megaleranthis saniculifolia	AY515399	NC_012615	NC_008796	NC_008796	NC_008796	NC_012615
Halerpestes cymbalaria	AY680196	FJ639873			AY954237	
Ficaria verna	AY680192	FJ639874			EU053919	
Anemone hupehensis	Unpubl.	Unpubl.			FJ626488	
Anemoclema glaucifolium	Unpubl.	Unpubl.			FJ626487	

**8. *Ranunculus***		ITS	*matK*
	Ranunculus aberdaricus		EU288371
	Ranunculus abortivus	AY680048	AY954126
	Ranunculus acaulis	AF323319	
	Ranunculus acetosellifolius	AY680075	AY954226

Ranunculus aconitifolius	AY680081	AY954217
Ranunculus acriformis	HQ338296	HQ338378
Ranunculus acris	AY680167	AY954199
Ranunculus adoneus	AY680030	FM242765
Ranunculus adoxifolius	JF509959	JF509970
Ranunculus aduncus	AY680088	AY954143
Ranunculus affinis	FM242811	FM242747
Ranunculus afghanicus	HQ338297	HM565146
Ranunculus alismifolius	HQ338298	HM565147
Ranunculus allegheniensis	JF509960	JF509971
Ranunculus allemannii	AY680039	JF509972
Ranunculus alpestris	AY680078	AY954221
Ranunculus altaicus	AY680112	AY954116
Ranunculus amblyolobus	HQ338299	HM565148
Ranunculus amerophyllus	AY680146	
Ranunculus ampelophyllus	FM242842	FM242778
Ranunculus amplexicaulis	AY680071	AY954223
Ranunculus amurensis	FM242820	FM242756
Ranunculus anadyriensis	FM242802	FM242738
Ranunculus anemoneus	AF323273	
Ranunculus apenninus	AY680091	AY954150
Ranunculus apiifolius	AY680092	AY954140
Ranunculus aquatilis	FM242843	FM242779
Ranunculus arcticus	AY680049	AY954125
Ranunculus argyreus	FM242844	FM242780
Ranunculus arvensis	HQ650550	HQ650551
Ranunculus ashibetsuensis		AB296104
Ranunculus asiaticus	GU257963	GU257985
Ranunculus aucheri	HQ338301	HQ338379
Ranunculus auricomus	FM242803	FM242739
Ranunculus baldshuanicus	AY680174	AY954195
Ranunculus basilobatus	AY680131	
Ranunculus baudotii	FM242858	FM242794
Ranunculus bequaertii	EU288399	EU288372
Ranunculus bilobus	AY680077	AY954220
Ranunculus biternatus	AY680061	
Ranunculus bonariensis		GU257986
Ranunculus borealis	AY680168	FM242766
Ranunculus brachylobus	HQ338302	HQ338347
Ranunculus brassii	AY680127	
Ranunculus brevifolius	AY680187	AY954212
Ranunculus breyninus	AY680116	AY954172
Ranunculus brotherusii	AY680037	AY954119
Ranunculus brutius	HQ338304	HQ338348

Ranunculus buchanani	AF323280	
Ranunculus bulbosus	AY680124	AY954188
Ranunculus bullatus	AY680114	AY954161
Ranunculus cacuminis	HQ338305	HQ338373
Ranunculus californicus	FM242846	FM242782
Ranunculus cantoniensis	HQ338306	HM565150
Ranunculus canus	FM242847	FM242783
Ranunculus cappadocicus	AY680117	AY954173
Ranunculus caprarum	AY680151	HM565151
Ranunculus cardiophyllus	AY680045	AY954124
Ranunculus carinthiacus	AY680093	AY954145
Ranunculus carpaticola	AY680041	AY954111
Ranunculus carpaticus	AY680096	AY954154
Ranunculus carpinetorum	AY680031	
Ranunculus cassius	FM242848	FM242784
Ranunculus cassubicifolius	AY680040	AY954112
Ranunculus cassubicus	FM242821	FM242757
Ranunculus caucasicus	AY680178	AY954192
Ranunculus cf. buhsei	FM242860	FM242796
Ranunculus cf. pseudopygmaeus	HQ338314	HQ338374
Ranunculus chamissonis	AY680083	AY954218
Ranunculus cheirophyllus	GU257965	GU257987
Ranunculus chilensis	AY680157	AY954179
Ranunculus chinensis	HQ338307	HQ338349
Ranunculus chius	AY680176	AY954201
Ranunculus cicutarius	AY680103	AY954167
Ranunculus circinatus	HQ894454	JN895832
Ranunculus collinus	AY680059	AY954137
Ranunculus colonorum	AY680139	
Ranunculus constantinopolitanus	HQ338308	HQ338350
Ranunculus cornutus	AY680153	
Ranunculus cortusifolius	AY680101	AY954160
Ranunculus crassipes	AY680060	
Ranunculus crenatus	AY680086	AY954228
Ranunculus creticus	AY954239	AY954163
Ranunculus crithmifolius	AF323313	
Ranunculus cryptanthus		EU288373
Ranunculus cupreus	AY954240	AY954164
Ranunculus dahuricus	FM242823	FM242759
Ranunculus damascenus	HQ338309	HM565153
Ranunculus diffusus	HQ338310	HQ338351
Ranunculus dissectus	FM242849	FM242785
Ranunculus eichleranus	AY680138	
Ranunculus elbrusensis	HQ338311	HQ338352

Ranunculus enysii	AF323317	
Ranunculus eschscholtzii	AY680050	AY954127
Ranunculus fallax	FM242824	FM242760
Ranunculus fascicularis	HQ338312	FM242786
Ranunculus ficariifolius	HQ338313	HQ338375
Ranunculus flagelliformis	AY680182	AY954208
Ranunculus flammula	AY680185	AY954204
Ranunculus fluitans	AY680069	AY954129
Ranunculus formosa-montanus	GU257966	GU257988
Ranunculus fuegianus	AY680064	AY954136
Ranunculus garganicus	AY680107	AY954165
Ranunculus gayeri	AY680028	
Ranunculus gelidus	AY680054	AY954114
Ranunculus glaberrimus	JF509962	JF509974
Ranunculus glabriusculus	FM242812	FM242748
Ranunculus glacialis	AY680082	AY954219
Ranunculus gmelinii	AY680063	AY954128
Ranunculus godleyanus	AF323309	
Ranunculus gouanii	AY680098	AY954151
Ranunculus gracilipes	AF323315	
Ranunculus gracilis	AY680120	AY954171
Ranunculus grahami	AF323287	
Ranunculus gramineus	AY680076	AY954227
Ranunculus granatensis	AY680165	AY954197
Ranunculus grandiflorus	AY680053	AY954203
Ranunculus grandifolius	AY680169	FM242772
Ranunculus graniticola	AY680141	
Ranunculus gregarius	AY680100	AY954159
Ranunculus gunnianus	AF323298	
Ranunculus haastii	AF323301	
Ranunculus hawaiensis	HQ338316	HM565155
Ranunculus hederaceus		JN895320
Ranunculus heterorrhizus	HQ338317	HM565156
Ranunculus hierosolymitanus	HQ338318	HQ338354
Ranunculus hirtellus	AY680038	AY954120
Ranunculus hispidus	HQ338319	HQ338355
Ranunculus hungaricus	FJ619889	FJ625807
Ranunculus hybridus	AY680189	AY954211
Ranunculus hydrophilus	AY680181	HM565157
Ranunculus hyperboreus	AY680065	AY954135
Ranunculus illyricus	AY680119	AY954162
Ranunculus inamoenus	FM242851	FM242787
Ranunculus induratus	AY680125	
Ranunculus insignis	AF323306	AY954141

Ranunculus japonicus	AY680164	AY954200
Ranunculus jovis	JF509963	JF509975
Ranunculus junipericola	JF509964	JF509976
Ranunculus kadzusensis		AB296112
Ranunculus kotschyi	HQ338320	HQ338356
Ranunculus krylovii	FM242826	FM242762
Ranunculus kuepferi	AY680085	AY954213
Ranunculus laetus	AY680172	HM565158
Ranunculus lanuginosus	AY680163	AY954194
Ranunculus lappaceus	AY680140	
Ranunculus lasiocarpus	FM242813	FM242763
Ranunculus lateriflorus	AY680179	AY954209
Ranunculus leptorrhynchus	HQ338323	HQ338358
Ranunculus linearilobus	HQ338324	HQ338359
Ranunculus lingua	AY680184	AY954206
Ranunculus lomatocarpus		AY954178
Ranunculus longicaulis	AY680051	AY954117
Ranunculus lowii	AY680128	
Ranunculus lyallii	AF323283	AY954142
Ranunculus macauleyi	JF509965	JF509977
Ranunculus maclovianus	AY680158	AY954181
Ranunculus macounii	FM242828	FM242764
Ranunculus macranthus		NC_008796
Ranunculus macropodoides	HQ338326	HQ338360
Ranunculus macrorrhynchus	HQ338327	HM565160
Ranunculus magellensis	HQ338328	HQ338376
Ranunculus makaluensis	HQ338329	HM565161
Ranunculus marginatus	AY680150	AY954177
Ranunculus marschlinsii	AY680089	AY954147
Ranunculus mauiensis	HQ338330	HM565162
Ranunculus melzeri	AY680036	
Ranunculus membranaceus	HQ338331	HQ338361
Ranunculus meyeri	EU288400	EU288374
Ranunculus micranthus	AY680042	AY954113
Ranunculus millanii	AY680134	
Ranunculus millefoliatus	AY680108	AY954166
Ranunculus millefolius		HQ338362
Ranunculus minutiflorus	AY680156	
Ranunculus monophyllus	AY680043	
Ranunculus montanus	AY680094	AY954149
Ranunculus muelleri	AY680143	
Ranunculus multifidus	EU288401	EU288375
Ranunculus multiscapus	AY680133	
Ranunculus muricatus	AY680148	AY954191

Ranunculus nanus	AY680142	
Ranunculus natans	AY680113	AY954134
Ranunculus neapolitanus	AY680123	AY954187
Ranunculus nephelogenes	HQ338333	HQ338363
Ranunculus niphophilus	AY680145	
Ranunculus nipponicus	FM242834	FM242770
Ranunculus nivalis	AY680046	AY954123
Ranunculus nivicola	AF323308	
Ranunculus notabilis	AY680033	AY954115
Ranunculus novus	FM242833	FM242769
Ranunculus occidentalis	HQ338334	HM565164
Ranunculus ollissiponensis	AY680109	AY954157
Ranunculus omiophyllus		JN895679
Ranunculus ophioglossifolius	AY680180	AY954207
Ranunculus oreophytus	EU288411	EU288385
Ranunculus orthorhynchus	HQ338335	HQ338364
Ranunculus oxyspermus	FM242863	FM242799
Ranunculus pachyrrhizus	AF323295	
Ranunculus palmatifidus	JF509978	JF509966
Ranunculus paludosus	AY680102	AY954155
Ranunculus pannonicus	AY680032	JF509979
Ranunculus papulentus	AY680058	AY954138
Ranunculus papyrocarpus	GU257968	GU257990
Ranunculus parnassifolius	AY680072	AY954224
Ranunculus parviflorus	AY680175	AY954202
Ranunculus pedatifidus	FM242808	FM242744
Ranunculus peduncularis	AY680154	AY954180
Ranunculus pegaeus	JF509967	JF509980
Ranunculus peltatus	AY680068	AY954131
Ranunculus penicillatus	AY680070	AY954130
Ranunculus pensylvanicus	AY680147	AY954190
Ranunculus petiolaris	HQ338336	HQ338365
Ranunculus pilisiensis	AY680034	JF509981
Ranunculus pimpinellifolius	AY680136	
Ranunculus pinardi	GU257970	GU257992
Ranunculus pinguis	AF323299	
Ranunculus pinnatus	EU288415	EU288388
Ranunculus platanifolius	AY680080	AY954216
Ranunculus plebeius	AY680137	
Ranunculus polii	DQ410717	
Ranunculus pollinensis	AY680097	AY954152
Ranunculus polyanthemoides	FM242865	FM242801
Ranunculus polyanthemos	AY680121	AY954185
Ranunculus polyphyllus	FM242838	FM242774

Ranunculus polyrhizos	FM242839	FM242775
Ranunculus praemorsus	AY680161	
Ranunculus prasinus	AY680057	
Ranunculus propinquus	AY680170	
Ranunculus pseudohirculus	AY680111	AY954118
Ranunculus pseudolowii	AY680130	
Ranunculus pseudomillefoliatus	AY680110	AY954156
Ranunculus pseudomontanus	AY680090	AY954146
Ranunculus pseudotrullifolius	AY680203	AY954139
Ranunculus psilostachys	AY680106	AY954170
Ranunculus pulchellus	FM242856	FM242792
Ranunculus punctatus	FM242818	FM242754
Ranunculus pygmaeus	AY954242	AY954122
Ranunculus pyrenaeus	AY680074	AY954225
Ranunculus radicans	FM242857	FM242793
Ranunculus raeae	EU288416	EU288389
Ranunculus recens	AF323320	
Ranunculus recurvatus	AY680118	AY954175
Ranunculus regelianus	HQ338338	HQ338366
Ranunculus repens	AY680160	AY954182
Ranunculus reptabundus	FM242819	FM242755
Ranunculus reptans	AY680186	AY954205
Ranunculus rhomboideus	FM242854	FM242790
Ranunculus rigescens	FM242809	FM242745
Ranunculus rionii	FM242855	FM242791
Ranunculus rubrocalyx	JF509968	JF509982
Ranunculus rufosepalus	AY680047	AY954121
Ranunculus rumelicus	AY680104	AY954168
Ranunculus sabinei	FM242815	FM242751
Ranunculus sardous	AY680122	AY954186
Ranunculus sartorianus	AY680095	AY954148
Ranunculus saruwagedicus	AY680129	
Ranunculus scapigerus	AY680135	
Ranunculus sceleratus	GU257971	GU257993
Ranunculus scrithalis	AF323305	
Ranunculus seguieri	EU792856	AY954215
Ranunculus septentrionalis	FM242832	FM242768
Ranunculus serbicus	AY680166	AY954196
Ranunculus sericeus	HQ338340	HM565167
Ranunculus sericocephalus	AY680155	
Ranunculus sericophyllus	AF323291	
Ranunculus serpens ssp. *nemorosus*	AY954243	AY954184
Ranunculus shuichengensis	DQ410719	
Ranunculus sieboldii	DQ410726	

Ranunculus silerifolius	HQ338341	HQ338367
Ranunculus simensis	EU288418	EU288391
Ranunculus sojakii	HQ338342	HQ338368
Ranunculus sphaerospermus	AY680066	AY954132
Ranunculus spicatus	AY954244	AY954158
Ranunculus sprunerianus	AY680105	AY954169
Ranunculus stagnalis	EU288419	EU288392
Ranunculus steveni	FM242864	FM242800
Ranunculus strigillosus	HQ338343	HQ338369
Ranunculus subcorymbosus	FM242810	FM242746
Ranunculus submarginatus	FM242841	FM242777
Ranunculus subscaposus	AY680132	
Ranunculus sulphureus	FM242816	FM242752
Ranunculus taisanensis	HQ338344	HQ338370
Ranunculus tanguticus	AY680055	
Ranunculus tembensis	EU288421	EU288393
Ranunculus tenuirostris	HQ338345	HQ338371
Ranunculus termei	HQ338346	HQ338372
Ranunculus ternatus	DQ410716	
Ranunculus thora	AY680188	AY954210
Ranunculus traunfellneri	AY954245	AY954222
Ranunculus trichophyllus	AY680067	AY954133
Ranunculus trigonus	DQ410724	
Ranunculus trilobus	AY680149	AY954176
Ranunculus tripartitus		JN895146
Ranunculus trullifolius	AY680159	
Ranunculus turneri	FM242805	FM242753
Ranunculus uncinatus	GU257972	GU257994
Ranunculus vaginatus	DQ410720	
Ranunculus variabilis	AY680029	
Ranunculus velutinus	AY680173	AY954198
Ranunculus venetus	AY680087	AY954144
Ranunculus verticillatus	AF323303	
Ranunculus villarsii	AY680099	AY954153
Ranunculus vindobonensis	AY680035	JF509984
Ranunculus viridis	AF323297	
Ranunculus volkensii	EU288424	EU288396
Ranunculus weyleri	AY954246	AY954189
Ranunculus yezoensis		AB296108
Laccopetalum giganteum	EU053931	DQ400695
Krapfia clypeata	GU552271	DQ490058
Ceratocephala falcata	AY680191	GU257996
Ceratocephala orthoceras	AY680190	AY954230
Coptidium pallasii	AY680195	AY954233

	Ficaria verna	AY680192	AY954232			
	Myosurus minimus	AJ347913	FJ626502			
9. ***Thalictrum***		ITS	*rbcL*	*trnL-F*	*ndhA*	*rpl32-trnL*
	Thalictrum actaeifolium	JX233660	JX258329	JX573432	JX258544	JX258436
	Thalictrum acutifolium	JX233758	JX258423	JX573521	JX258637	JX258529
	Thalictrum alpinum	JX233664	JX258333	JX573436	JX258548	JX258440
	Thalictrum aquilegiifolium	JX233666	JX258335	JX573438	JX258550	JX258442
	Thalictrum arkansanum	JX233669	JX258338	JX573441	JX258553	JX258445
	Thalictrum arsenii	JX233670	JX258339	JX573442	JX258554	JX258446
	Thalictrum atriplex	JX233759	JX258424	JX573522	JX258638	JX258530
	Thalictrum baicalense	JX233672	JX258341	JQ691506	JX258555	JX258448
	Thalictrum brevisericeum	JX233673				JX258449
	Thalictrum calabricum	JX233674	JX258342	JX573443	JX258556	JX258450
	Thalictrum calcicola	JX233766	JX258429	JX573528	JX258644	JX258537
	Thalictrum chayuense	JX233760				JX25853
	Thalictrum chelidonii	JX233761	JX258425	JX573523	JX258639	JX258532
	Thalictrum cirrhosum	JX233762		JX573524	JX258640	JX258533
	Thalictrum clavatum	JX233675	JX258343	JX573444	JX258557	JX258451
	Thalictrum confine	JX233676	JX258344	JX573445	JX258558	JX258452
	Thalictrum cooleyi	JF742136				
	Thalictrum coreanum	JX233678	JX258346	JX573447	JX258560	JX258454
	Thalictrum coriaceum	JX233680	JX258348	JX573449	JX258562	JX258456
	Thalictrum cultratum	JX233763	JX258426	JX573525	JX258641	JX258534
	Thalictrum dasycarpum	JX233682	JX258350	JX573451	JX258564	JX258458
	Thalictrum decipiens	JX233683	JX258351	JX573452	JX258565	JX258459
	Thalictrum delavayi	JX233684	JX258352	JX573453	JX258566	JX258460
	Thalictrum diffusiflorum	JF742144				
	Thalictrum dioicum	JX233686	JX258354	JX573455	JX258568	JX258462
	Thalictrum elegans	JX233687	JX258355	JX573456	JX258569	JX258463
	Thalictrum fargesii	JX233688	JX258356	JX573457	JX258570	JX258464
	Thalictrum fendleri	JX233690	JX258358	JX573459	JX258572	JX258466
	Thalictrum filamentosum	JF742148				
	Thalictrum finetii	JX233764	JX258427	JX573526	JX258642	JX258535
	Thalictrum flavum	JF742162	JN892776			
	Thalictrum foeniculaceum	JX233691	JX258359	JX573460	JX258573	JX258467
	Thalictrum foetidum	JX233692	JX258360	JX573461	JX258574	JX258468
	Thalictrum foliolosum	JX233693	JX258361	JX573462	JX258575	JX258469
	Thalictrum galeottii	JX233694	JX258362	JX573463	JX258576	JX258470
	Thalictrum gibbosum	JX233695	JX258363	JX573464	JX258577	JX258471
	Thalictrum grandiflorum	JX233696	JX258364	JX573465	JX258578	JX258472
	Thalictrum grandisepalum	JX233730	JX258396	JX573495	JX258610	JX258504
	Thalictrum guatemalense	JX233698	JX258366	JX573467	JX258580	JX258474
	Thalictrum heliophilum	JF742154				
	Thalictrum henricksonii	JF742155				

Thalictrum hernandezii	JX233699	JX258367	JX573468	JX258581	JX258475
Thalictrum ichangense	JX233700	JX258368	JX573469	JX258582	JX258476
Thalictrum isopyroides	JF742158				
Thalictrum javanicum	EF437124	AY954496	EF437107		
Thalictrum kiusianum	JF742159				
Thalictrum lankesteri	JX233701	JX258369	JX573470	JX258583	JX258477
Thalictrum lecoyeri	JX233765	JX258428	JX573527	JX258643	JX258536
Thalictrum leuconotum	JX233702	JX258370	JX573471	JX258584	JX258478
Thalictrum lucidum	JX233703	JX258371	JX573472	JX258585	JX258479
Thalictrum macrocarpum	JX233704	JX258372	JX573473	JX258586	JX258480
Thalictrum macrostylum	EU438837				
Thalictrum minus	JF742164	EU053923	GQ245606	KC288714	KC290224
Thalictrum myriophyllum	JX233706	JX258374	JX573474	JX258588	JX258482
Thalictrum occidentale	JX233707	JX258375	JX573475	JX258589	JX258483
Thalictrum omeiense	JX233709	JX258377	JX573477	JX258591	JX258485
Thalictrum osmorhizoides	JX233710		JQ691523		
Thalictrum peltatum	JX233711	JX258378	JX573478	JX258592	JX258486
Thalictrum petaloideum	JX233712	JX258379	JX573479	JX258593	JX258487
Thalictrum pinnatum	JX233714	JX258381	JX573481	JX258595	JX258489
Thalictrum podocarpum	JF742169				
Thalictrum polycarpum	JX233716	JX258383	JX573483	JX258597	JX258491
Thalictrum pringlei	JX233717	JX258384	JX573484	JX258598	JX258492
Thalictrum przewalskii	JX233718	JX258385	JX573485	JX258599	JX258493
Thalictrum pubescens	JX233721	JX258388	JX573488	JX258602	JX258496
Thalictrum pubigerum	JX233722	JX258389	JX573489	JX258603	JX258497
Thalictrum punctatum	JX233723	JX258390	JQ691528	JX258604	JX258498
Thalictrum ramosum	JF742173				
Thalictrum reniforme	JX233725	JX258391	JX573490	JX258605	JX258499
Thalictrum revolutum	JX233726	JX258392	JX573491	JX258606	JX258500
Thalictrum rhynchocarpum	JX233728	JX258394	JX573493	JX258608	JX258502
Thalictrum robustum	EF437125	EF437148	EF437108		
Thalictrum rochebrunnianum	JX233729	JX258395	JX573494	JX258609	JX258503
Thalictrum rostellatum	JX233731	JX258397	JX573496	JX258611	JX258505
Thalictrum rotundifolium	JX233732	JX258398	JX573497	JX258612	JX258506
Thalictrum rubescens	JX233733	JX258399	JX573498	JX258613	
Thalictrum rutifolium	JX233734	JX258400	JX573499	JX258614	JX258507
Thalictrum sachalinense	JX233735	JX258401	JX573500	JX258615	JX258508
Thalictrum saniculiforme	JX233736	JX258402	JX573501	JX258616	JX258509
Thalictrum simplex	JF742181	FJ449863			
Thalictrum smithii	JX233738	JX258404	JX573503	JX258618	JX258511
Thalictrum sparsiflorum	JX233739	JX258405	JX573504	JX258619	JX258512
Thalictrum squamiferum	JX233741	JX258407	JX573505	JX258621	JX258514
Thalictrum squarrosum	JX573506	JX258408	JX573506	JX258622	JX258515
Thalictrum steyermarkii	JF742186				

Thalictrum strigillosum	JX233744	JX258409	JX573507	JX258623	JX258516
Thalictrum taqueti			JQ691505		
Thalictrum tenue	JX233745	JX258410	JX573508	JX258624	JX258517
Thalictrum texanum	JX233747	JX258412	JX573510	JX258626	JX258519
Thalictrum thalictroides	JX233748	EU053924	JX573511	JX258627	
Thalictrum trichopus	JX233749	JX258414	JX573512	JX258628	JX258520
Thalictrum tripeltiferum	JF742188				
Thalictrum tuberiferum	JX233750	JX258415	JX573513	JX258629	JX258521
Thalictrum tuberosum	JX233751	JX258416	JX573514	JX258630	JX258522
Thalictrum uchiyamae	JX233752	JX258417	JX573515	JX258631	JX258523
Thalictrum uncatum	JX233753	JX258418	JX573516	JX258632	JX258524
Thalictrum uncinulatum	JX233754	JX258419	JX573517	JX258633	JX258525
Thalictrum urbainii	JF742146				
Thalictrum venulosum	JX233756	JX258421	JX573519	JX258635	JX258527
Thalictrum virgatum	JX233757	JX258422	JX573520	JX258636	JX258528
Thalictrum zernyi	JF742193				
Paropyrum anemonoides	EF437118	EF437142	EF437101	KC288716	KC290226
Enemion raddeanum	EF437117	AY954494	EF437100	JX258645	JX258538
Paraquilegia microphylla	EF437122	EF437146	EF437105	JX258649	JX258542
Semiaquilegia adoxoides	EF437123	EF437147	EF437106	JX258648	JX258541
Leptopyrum fumarioides	EF437121	EF437145	EF437104	JX258650	

Supplementary Methods

Phylogenetic analysis. We first used RAxML v7.0.4[21] to perform nonparametric bootstrap analysis for each marker. No bootstrap support for conflicting nodes was significant (taken here as exceeding 70%), and data from different makers were therefore combined for subsequent analyses.

Calibration points. Following the methods reviewed by Sauquet *et al.*[22], we selected three buttercup fossils as internal calibration points as follows.

(1) A fossil eudicot genus with reproductive organs, *Leefructus,* was reported from the Lower Cretaceous Yixian Formation at 125.8 to 122.6 Ma, and was regarded as a stem lineage of the extant family Ranunculaceae[23]. Molecular and morphological analyses of extant Ranunculales combined with *Leefructus* also indicate that it has an affinity with the extant Ranunculaceae[24]. Thus, we constrained the stem age of the extant Ranunculaceae using a minimum age of 122.6 Ma.

(2) The extinct genus *Paleoactea* contains two species, *P. nagelii* and *P. bowerbanki*, which was found in the Late Paleocene Almont and Beicegel Creek floras of North Dakota, USA, and the Early Eocene London Clay flora of southern England, respectively[25]. Based on fruit size and shape and details of seed morphology and anatomy, *Paleoactea* is markedly close to the extant *Actaea*. Nevertheless, *Paleoactea* and *Cimicufuga* share the presence of scales on the seeds. Additionally, the flattened luniform seeds occur in *Actaea* and four species of *Cimicifuga* (*C. racemosa*, *C. bitemata*, *C. japonica*, and *C. purpurea*). Compton *et al.*[12]

circumscribed *Actaea* in a broad sense to include also *Cimicifuga* and *Souliea*. Thus, we conservatively placed *Paleoactea* on the lineage leading to the extant, broadly defined *Actaea*, and used the Eocene/Paleocene boundary (56.0 Ma[26]) as the lower bound of the split of the genus.

(3) Fossil achenes of *Myosurus* were found from the Oligocene[27]. Achenes of *Myosurus* have so unique morphological and anatomical features within the Ranunculeae[28] that the fossil determination of the genus can be considered to be confident[29]. We thus used the Miocene/Oligocene boundary (23.03 Ma[26]) as the lower bound of the split of the genus.

Supplementary divergence time estimates. Based on divergence time estimates from the 88-taxon data set, our taxon sampling could be incomplete for five candidate lineages, Caltheae, Callianthemeae, Helleboreae, Nigelleae, and *Eranthis* by 47.8 Ma (end of Ypresian). In order to determine the crown age for each of these lineages, we assembled four supplementary data sets comprising the following DNA regions: Caltheae and Callianthemeae (ITS, *trnL-F* and *atpB-rbcL*), Helleboreae (ITS, *trnL-F* and *matK*), Nigelleae (ITS), and *Eranthis* (ITS and *trnL-F*). Supplementary molecular dating analyses were performed with overlapping nodes from the 88-taxon data set as calibration points with normal distributions. Taxon sampling and accession numbers for supplementary molecular dating analyses are listed in Supplementary Table 7. These supplementary molecular dating analyses indicate that all five lineages diversified after 47.8 Ma (Supplementary Table 4), which suggests that our taxon

sampling is complete at 47.8 Ma.

Ancestral state reconstructions. Thirteen genera in Ranunculaceae inhabit various habitats, *Aconitum, Adonis, Anemone, Aquilegia, Calathodes, Caltha, Clematis, Delphinium, Helleborus, Hepatica, Ranunculus, Thalictrum,* and *Trollius*. In order to determine the habitat state for each of their most recent common ancestors, we assembled eight supplementary data sets comprising the following DNA regions: Adonideae (including *Adonis*, and *Calathodes*; ITS, *trnL-F* and *matK*), Anemoneae (focusing on *Anemone* and *Hepatica*; ITS, *atpB-rbcL, matK*, and *rbcL*), *Caltha* (ITS, *trnL-F* and *atpB-rbcL*), *Clematis* (ITS, *atpB-rbcL, psbA-trnQ, rpoB-trnC, trnK-matK*, and *rbcL–accD*), *Helleborus* (ITS, *trnL-F* and *matK*), *Ranunculus* (ITS and *matK*), and *Thalictrum* (ITS, *rbcL, trnL-F, ndhA*, and *rpl32-trnL*). Taxon sampling and accession numbers for supplementary ancestral habitat reconstructions are listed in Supplementary Table 7. Data sets for *Aquilegia* and Delphinideae are from Fior *et al.*[30] and Wang *et al.*[3], respectively. We first performed RAxML analyses to find ML tree for each of the nine supplementary data sets. We then inferred ancestral states for these lineages using the maximum likelihood method in Mesquite v. 2.75[31]. After outgroups were excluded, analyses were carried out on the ML trees, taking into account branch lengths, and using the Markov k-state one-parameter model. This approach is a generalization of the Jukes-Cantor model[32] and assumes a single rate for all transitions between character states. Habitat data were obtained from the taxonomic literature, herbarium records, and personal field observations. The inferred results are indicated in supplementary Table 1.

Diversification analyses. In this study, our taxon sampling is complete before 47.8 Ma (Supplementary Table 4). In order to avoid potential effects of nonsampled taxa on diversification rate analyses, we here focused on the time period between the estimated origin of Ranunculaceae and 47.8 Ma. The BAMM is a recent developed software, which fits four diversification models (time-dependent, diversity-dependent, and constant rate pure birth or birth-death) to ultrametric trees[33]. However, its power to detect rate variation decreases in small trees although the program works well with trees as small as 87 terminals[33]. Nevertheless, Ranunculaceae only included 13 extant lineages before 47.8 Ma. The MEDUSA is a maximum likelihood method for modeling among-lineage heterogeneity in speciation-extinction dynamics[34]. Rabosky[33] suggests that MEDUSA often underestimates the true number of processes in simulated datasets when rates of speciation vary over time and that branch-specific speciation rates evaluated with MEDUSA show little correspondence with true rates. In this study, we first generated standard lineages-through-time plots in APE v3.1[35]. Net diversification rates were calculated using TreePar[36] and Geiger v1.99-3[37]. Additionally, using a phylogenetically unstructured analysis[38], net diversification rates were used to compare the differences between forest-dwelling and open-vegetation clades (at the subfamilial and tribal levels, all of which occurred prior to 47.8 Ma).

Supplementary References

1. Tamura, M. 1995. Systematic part. *Natürliche Pflanzenfamilien*, 17a IV [Hiepko, P. (ed)] [223–497] (Duncker and Humblot, Berlin, 1995).

2. Wang, W., Lu, A. M., Ren, Y., Endress, M. E. & Chen, Z. D. Phylogeny and classification of Ranunculales: Evidence from four molecular loci and morphological data. *Perspect. Plant Ecol. Evol. Syst.* **11,** 81–110 (2009).
3. Wang, W., Liu, Y., Yu, S. X., Gao, T. G. & Chen, Z. D. *Gymnaconitum*, a new genus of Ranunculaceae endemic to the Qinghai–Tibetan Plateau. *Taxon* **62,** 713–722 (2013).
4. Bastida, J. M., Alcántara, J. M., Rey, P. J., Vargas, P. & Herrera, C. M. Extended phylogeny of Aquilegia: the biogeographical and ecological patterns of two simultaneous but contrasting radiations. *Plant Syst. Evol.* **284,** 171–185 (2010).
5. Wikström, N., Savolainen, V. & Chase, M. Evolution of angiosperms: calibrating the family tree. *Proc. Roy. Soc. B* **268,** 2211–2222 (2001).
6. Jabbour, F. & Renner, S. A phylogeny of Delphinieae (Ranunculaceae) shows that *Aconitum* is nested within *Delphinium* and that Late Miocene transitions to long life cycles in the Himalayas and southwest China coincide with bursts in diversification. *Mol. Phylogen. Evol.* **62,** 928–942 (2012).
7. Jabbour, F. & Renner, S. *Consolida* and *Aconitella* are an annual clade of *Delphinium* (Ranunculaceae) that diversified in the Mediterranean basin and the Irano-Turanian region. *Taxon* **60,** 1029–1040 (2011).
8. Emadzade, K. & Hörandl, E. Northern Hemisphere origin, transoceanic dispersal, and diversification of Ranunculeae DC. (Ranunculaceae) in the Cenozoic. *J. Biogeogr.* **38,** 517–530 (2011).
9. Wang, W., Li, H. L., Xiang, X. G. & Chen, Z. D. Revisiting the phylogeny of

Ranunculeae: Implications for divergence time estimation and historical biogeography. *J. Syst. Evol.* **52,** 551–565 (2014).

10. Anderson, C. L., Bremer, K. & Friis, E. M. Dating phylogenetically basal eudicots using *rbcL* sequences and multiple fossil reference points. *Am. J. Bot.* **92,** 1737–1748 (2005).
11. Xie, L., Wen, J. & Li, L. Q. Phylogenetic analyses of *Clematis* (Ranunculaceae) based on sequences of nuclear ribosomal ITS and three plastid regions. *Syst. Bot.* **36,** 907–921 (2011).
12. Compton, J. A., Culham, A. & Jury, S. L. Reclassification of *Actaea* to include *Cimicifuga* and *Souliea* (Ranunculaceae): phylogeny inferred from morphology, nrDNA ITS, and cpDNA *trnL-F* sequence variation. *Taxon* **47,** 59–634 (1998).
13. Hoot, S. B., Meyer, K. M. & Manning, J. C. 2012. Phylogeny and reclassification of *Anemone* (Ranunculaceae), with an emphasis on Austral species. *Syst. Bot.* **37,** 139–152.
14. Chen, Z. D. *et al.* Systematic position of the Rhoipteleaceae: Evidence from nucleotide sequences of the *rbcL* gene. *Acta Phytotax. Sin.* **36,** 1–7 (1998).
15. Wang, W., Chen, Z.D., Liu, Y., Li, R.Q. & Li, J.H. Phylogenetic and biogeographic diversification of Berberidaceae in the Northern Hemisphere. *Syst. Bot.* **32,** 731–742 (2007).
16. Hoot, S. B., Culham, A. & Crane, P. R. The utility of *atpB* gene sequences in resolving phylogenetic relationships: comparisons with *rbcL* and 18S ribosomal

DNA sequences in the Lardizabalaceae. *Ann. Missouri Bot. Gard.* **82,** 194–207 (1995).

17. Schuettpelz, E., Korall, P. & Pryer, K. M. Plastid *atpA* data provide improved support for deep relationships among ferns. *Taxon* **55,** 897–906 (2006).
18. Olmstead, R. G. & Sweere, J. A. 1994. Combining data in phylogenetic systematics: An empirical approach using three molecular data sets in the Solanaceae. *Syst. Bot.* 43, 467–481 (1994).
19. Wang, W. *et al.* Menispermaceae and the diversification of tropical rainforests near the Cretaceous–Paleogene boundary. *New Phytol.* **195,** 470–478 (2012).
20. Wang, W., Li, R. Q. & Chen, Z. D. Systematic position of *Asteropyrum* (Ranunculaceae) inferred from chloroplast and nuclear sequences. *Plant Syst. Evol.* **255,** 41–54 (2005).
21. Stamatakis, A. RAxML-VI-HPC: Maximum likelihood-based phylogenetic analyses with thousands of taxa and mixed models. *Bioinformatics* **22,** 2688–2690 (2006).
22. Sauquet, H. *et al.* 2012. Testing the impact of calibration on molecular divergence times using a fossil-rich group: the case of *Nothofagus* (Fagales). *Syst. Biol.* **61,** 289–313.
23. Sun, G., Dilcher, D. L., Wang, H. S. & Chen, Z. D. A eudicot from the Early Cretaceous of China. *Nature* **471,** 625–628 (2011).
24. Wang, W., Dilcher, D. L., Sun, G., Wang, H. S. & Chen, Z. D. Accelerated evolution of early angiosperms: Evidence from ranunculalean phylogeny by

integrating living and fossil data. *J. Syst. Evol.* DOI: 10.1111/jse.12090 (2014).

25. Pigg, K. B. & DeVore, M. L. 2005. *Paleoactaea* gen. nov (Ranunculaceae) fruits from the Paleogene of North Dakota and the London clay. *Am. J. Bot.* **92,** 1650–1659.
26. Cohen, K. M., Finney, S. C., Gibbard, P. L. & Fan, J. X. The ICS International Chronostratigraphic Chart. *Episodes* **36,** 199–204 (2013 updated).
27. Mai, D. H. & Walter, H. 1978. Die Floren der Haselbacher Serie im Weißelster-Becken (Bezirk Leipzig, DDR). *Abh. Staatl. Mus. Mineral. Geol. Dresden* **28,** 1–200 (1978).
28. Emadzade, K., Lehnebach, C., Lockhart, P. & Hörandl, E. A molecular phylogeny, morphology and classification of genera of Ranunculeae (Ranunculaceae). *Taxon* **59,** 809–828 (2010).
29. Emadzade, K. & Hörandl, E. Northern Hemisphere origin, transoceanic dispersal, and diversification of Ranunculeae DC. (Ranunculaceae) in the Cenozoic. *J. Biogeogr.* **38,** 517–530 (2011).
30. Fior, S. *et al.* Spatiotemporal reconstruction of the *Aquilegia* rapid radiation through nextgeneration sequencing of rapidly evolving cpDNA regions. *New Phytol.* **198,** 579–592 (2013).
31. Maddison, W. P. & Maddison, D. R. Mesquite: a modular system for evolutionary analysis, v1.12. Available at http://mesquiteproject.org (2011).
32. Lewis, P. O. A likelihood approach to estimating phylogeny from discrete morphological character data. *Syst. Biol.* **50,** 913–925 (2001).

33. Rabosky, D. L. Automatic detection of key innovations, rate shifts, and diversity dependence on phylogenetic trees. *PLoS ONE* **92,** e89543 (2014).

34. Alfaro, M. E. *et al.* Nine exceptional radiations plus high turnover explain species diversity in jawed vertebrates. *Proc. Natl. Acad. Sci. USA* **106,** 13410–13414 (2009).

35. Paradis, E., Claude, J. & Strimmer, K. APE: Analyses of phylogenetics and evolution in R language. *Bioinformatics* **20,** 289–290 (2004).

36. Stadler, T. Mammalian phylogeny reveals recent diversification rate shifts. *Proc. Natl. Acad. Sci. USA* **108,** 6187–6192 (2011).

37. Harmon, L. J., Weir, J. T., Brock, C. D., Glor, R. E. & Challenger, W. GEIGER: investigating evolutionary radiations. *Bioinformatics* **24,** 129–131 (2008).

38. Givnish, T. J. *et al.* Adaptive radiation, correlated and contingent evolution, and determinants of net species diversification in Bromeliaceae. *Mol. Phylogenet. Evol.* **71,** 55–78 (2014).

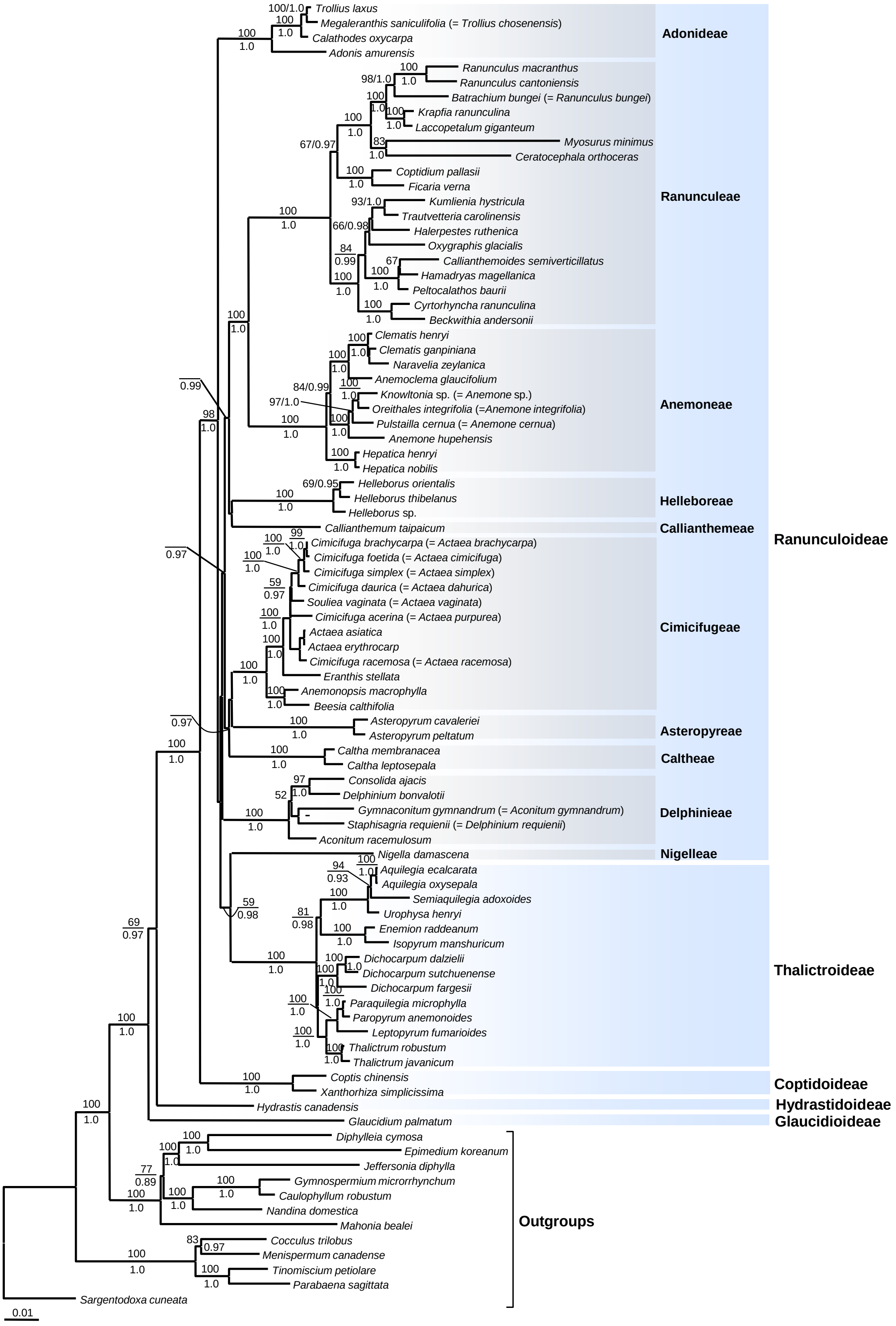

Trollius laxus
Megaleranthis saniculifolia (= Trollius chosenensis)
Calathodes oxycarpa
Adonis amurensis
Adonideae
Ranunculus macranthus
Ranunculus cantoniensis
Batrachium bungei (= Ranunculus bungei)
Krapfia ranunculina
Laccopetalum giganteum
Myosurus minimus
Ceratocephala orthoceras
Coptidium pallasii
Ficaria verna
Kumlienia hystricula
Trautvetteria carolinensis
Halerpestes ruthenica
Oxygraphis glacialis
Callianthemoides semiverticillatus
Hamadryas magellanica
Peltocalathos baurii
Cyrtorhyncha ranunculina
Beckwithia andersonii
Ranunculeae
Clematis henryi
Clematis ganpiniana
Naravelia zeylanica
Anemoclema glaucifolium
Knowltonia sp. (= Anemone sp.)
Oreithales integrifolia (=Anemone integrifolia)
Pulstailla cernua (= Anemone cernua)
Anemone hupehensis
Hepatica henryi
Hepatica nobilis
Anemoneae
Helleborus orientalis
Helleborus thibelanus
Helleborus sp.
Helleboreae
Callianthemum taipaicum
Callianthemeae
Cimicifuga brachycarpa (= Actaea brachycarpa)
Cimicifuga foetida (= Actaea cimicifuga)
Cimicifuga simplex (= Actaea simplex)
Cimicifuga daurica (= Actaea dahurica)
Souliea vaginata (= Actaea vaginata)
Cimicifuga acerina (= Actaea purpurea)
Actaea asiatica
Actaea erythrocarp
Cimicifuga racemosa (= Actaea racemosa)
Eranthis stellata
Anemonopsis macrophylla
Beesia calthifolia
Cimicifugeae
Asteropyrum cavaleriei
Asteropyrum peltatum
Asteropyreae
Caltha membranacea
Caltha leptosepala
Caltheae
Consolida ajacis
Delphinium bonvalotii
Gymnaconitum gymnandrum (= Aconitum gymnandrum)
Staphisagria requienii (= Delphinium requienii)
Aconitum racemulosum
Delphinieae
Nigella damascena
Nigelleae
Ranunculoideae
Aquilegia ecalcarata
Aquilegia oxysepala
Semiaquilegia adoxoides
Urophysa henryi
Enemion raddeanum
Isopyrum manshuricum
Dichocarpum dalzielii
Dichocarpum sutchuenense
Dichocarpum fargesii
Paraquilegia microphylla
Paropyrum anemonoides
Leptopyrum fumarioides
Thalictrum robustum
Thalictrum javanicum
Thalictroideae
Coptis chinensis
Xanthorhiza simplicissima
Coptidoideae
Hydrastis canadensis
Hydrastidoideae
Glaucidium palmatum
Glaucidioideae
Diphylleia cymosa
Epimedium koreanum
Jeffersonia diphylla
Gymnospermium microrrhynchum
Caulophyllum robustum
Nandina domestica
Mahonia bealei
Cocculus trilobus
Menispermum canadense
Tinomiscium petiolare
Parabaena sagittata
Sargentodoxa cuneata
Outgroups
0.01

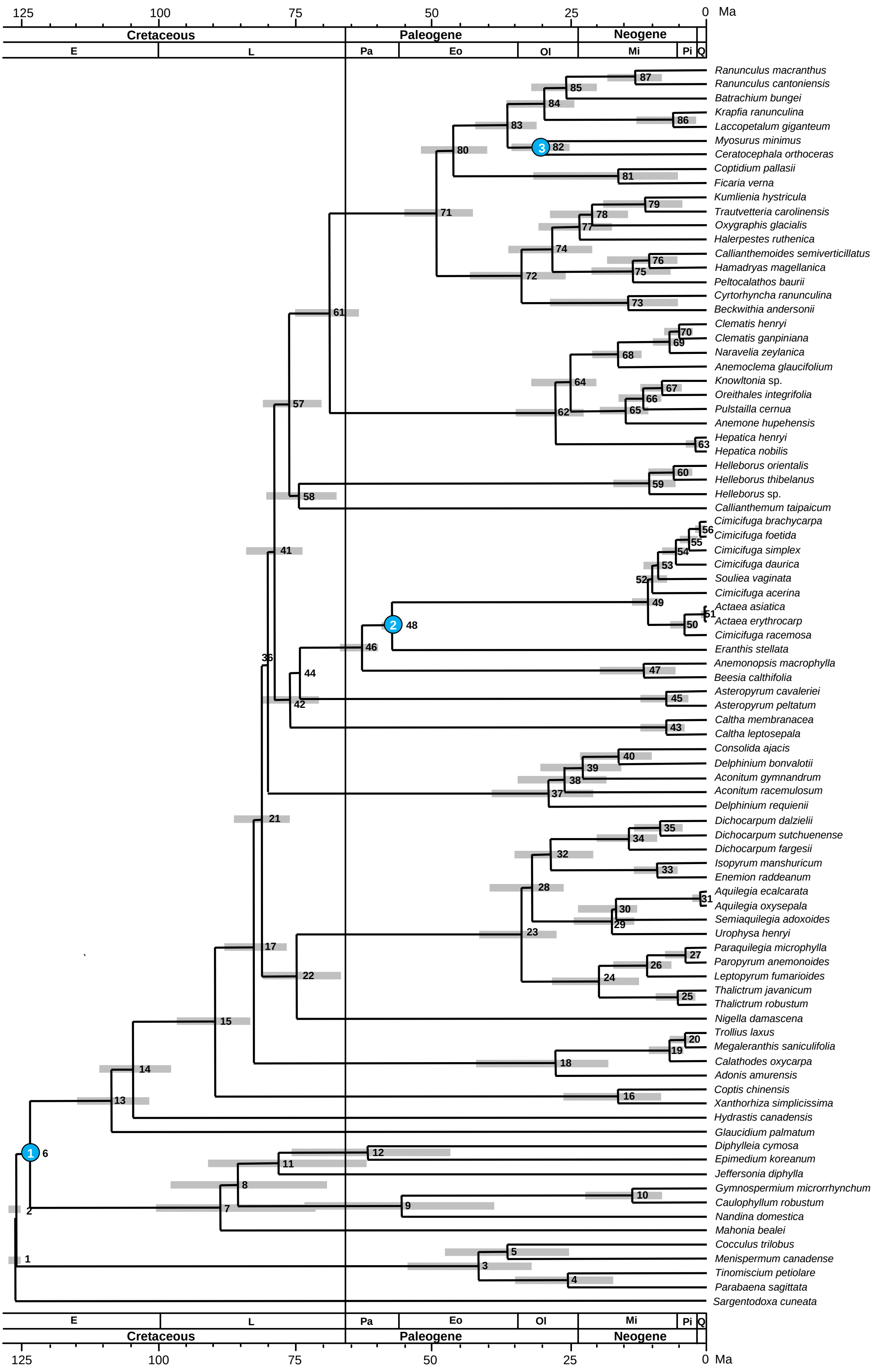
125 100 75 50 25 0 Ma
Cretaceous
Paleogene
Neogene
E
L
Pa
Eo
Ol
Mi
Pi
Q
Ranunculus macranthus
Ranunculus cantoniensis
Batrachium bungei
Krapfia ranunculina
Laccopetalum giganteum
Myosurus minimus
Ceratocephala orthoceras
Coptidium pallasii
Ficaria verna
Kumlienia hystricula
Trautvetteria carolinensis
Oxygraphis glacialis
Halerpestes ruthenica
Callianthemoides semiverticillatus
Hamadryas magellanica
Peltocalathos baurii
Cyrtorhyncha ranunculina
Beckwithia andersonii
Clematis henryi
Clematis ganpiniana
Naravelia zeylanica
Anemoclema glaucifolium
Knowltonia sp.
Oreithales integrifolia
Pulstailla cernua
Anemone hupehensis
Hepatica henryi
Hepatica nobilis
Helleborus orientalis
Helleborus thibelanus
Helleborus sp.
Callianthemum taipaicum
Cimicifuga brachycarpa
Cimicifuga foetida
Cimicifuga simplex
Cimicifuga daurica
Souliea vaginata
Cimicifuga acerina
Actaea asiatica
Actaea erythrocarp
Cimicifuga racemosa
Eranthis stellata
Anemonopsis macrophylla
Beesia calthifolia
Asteropyrum cavaleriei
Asteropyrum peltatum
Caltha membranacea
Caltha leptosepala
Consolida ajacis
Delphinium bonvalotii
Aconitum gymnandrum
Aconitum racemulosum
Delphinium requienii
Dichocarpum dalzielii
Dichocarpum sutchuenense
Dichocarpum fargesii
Isopyrum manshuricum
Enemion raddeanum
Aquilegia ecalcarata
Aquilegia oxysepala
Semiaquilegia adoxoides
Urophysa henryi
Paraquilegia microphylla
Paropyrum anemonoides
Leptopyrum fumarioides
Thalictrum javanicum
Thalictrum robustum
Nigella damascena
Trollius laxus
Megaleranthis saniculifolia
Calathodes oxycarpa
Adonis amurensis
Coptis chinensis
Xanthorhiza simplicissima
Hydrastis canadensis
Glaucidium palmatum
Diphylleia cymosa
Epimedium koreanum
Jeffersonia diphylla
Gymnospermium microrrhynchum
Caulophyllum robustum
Nandina domestica
Mahonia bealei
Cocculus trilobus
Menispermum canadense
Tinomiscium petiolare
Parabaena sagittata
Sargentodoxa cuneata

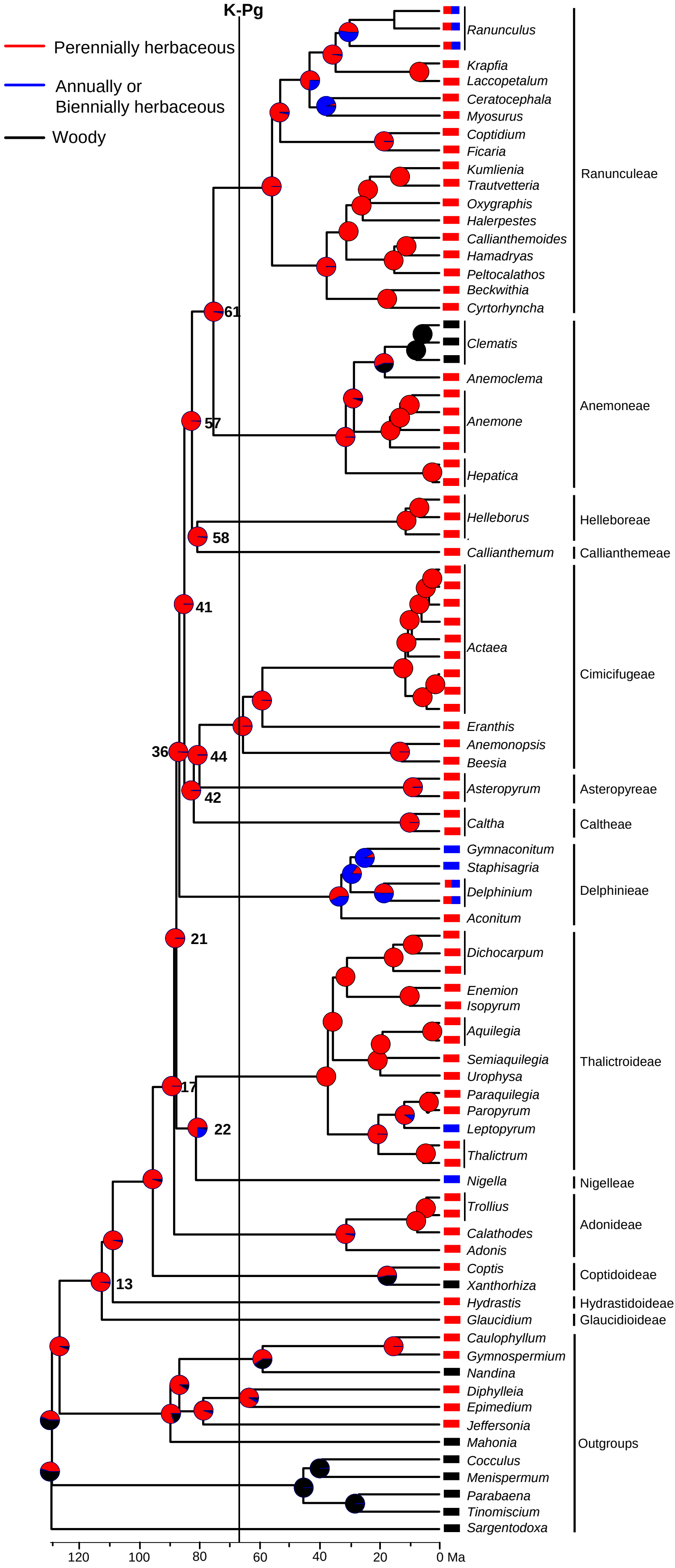
K-Pg
Perennially herbaceous
Annually or Biennially herbaceous
Woody
Ranunculus
Krapfia
Laccopetalum
Ceratocephala
Myosurus
Coptidium
Ficaria
Kumlienia
Trautvetteria
Oxygraphis
Halerpestes
Callianthemoides
Hamadryas
Peltocalathos
Beckwithia
Cyrtorhyncha
Ranunculeae
Clematis
Anemoclema
Anemone
Hepatica
Anemoneae
Helleborus
Helleboreae
Callianthemum
Callianthemeae
Actaea
Eranthis
Anemonopsis
Beesia
Cimicifugeae
Asteropyrum
Asteropyreae
Caltha
Caltheae
Gymnaconitum
Staphisagria
Delphinium
Aconitum
Delphinieae
Dichocarpum
Enemion
Isopyrum
Aquilegia
Semiaquilegia
Urophysa
Paraquilegia
Paropyrum
Leptopyrum
Thalictrum
Thalictroideae
Nigella
Nigelleae
Trollius
Calathodes
Adonis
Adonideae
Coptis
Xanthorhiza
Coptidoideae
Hydrastis
Hydrastidoideae
Glaucidium
Glaucidioideae
Caulophyllum
Gymnospermium
Nandina
Diphylleia
Epimedium
Jeffersonia
Mahonia
Cocculus
Menispermum
Parabaena
Tinomiscium
Sargentodoxa
Outgroups
61
57
58
41
36
44
42
21
17
22
13
120
100
80
60
40
20
0 Ma

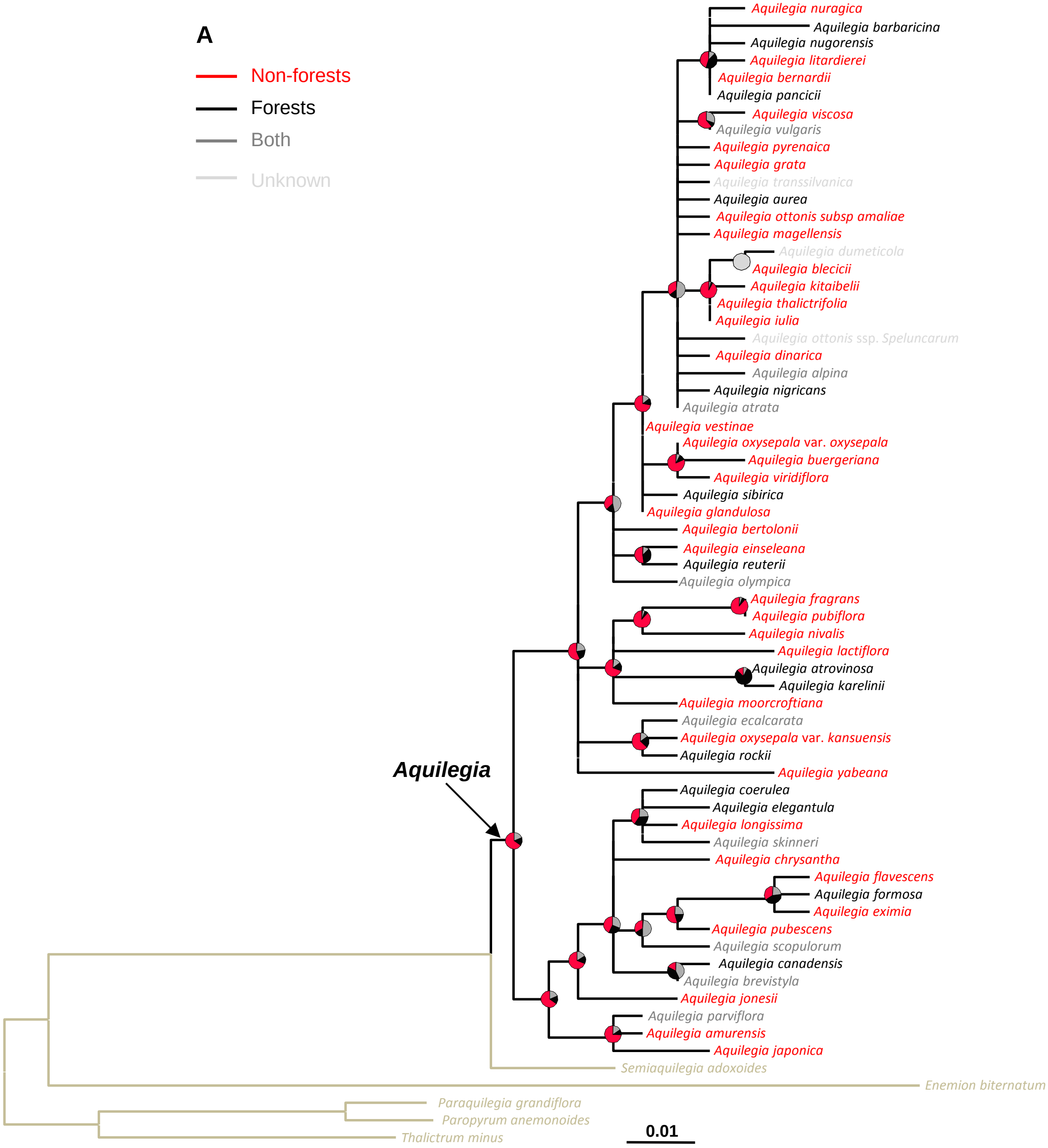
A
Non-forests
Forests
Both
Unknown
Aquilegia nuragica
Aquilegia barbaricina
Aquilegia nugorensis
Aquilegia litardierei
Aquilegia bernardii
Aquilegia pancicii
Aquilegia viscosa
Aquilegia vulgaris
Aquilegia pyrenaica
Aquilegia grata
Aquilegia transsilvanica
Aquilegia aurea
Aquilegia ottonis subsp amaliae
Aquilegia magellensis
Aquilegia dumeticola
Aquilegia blecicii
Aquilegia kitaibelii
Aquilegia thalictrifolia
Aquilegia iulia
Aquilegia ottonis ssp. Speluncarum
Aquilegia dinarica
Aquilegia alpina
Aquilegia nigricans
Aquilegia atrata
Aquilegia vestinae
Aquilegia oxysepala var. oxysepala
Aquilegia buergeriana
Aquilegia viridiflora
Aquilegia sibirica
Aquilegia glandulosa
Aquilegia bertolonii
Aquilegia einseleana
Aquilegia reuterii
Aquilegia olympica
Aquilegia fragrans
Aquilegia pubiflora
Aquilegia nivalis
Aquilegia lactiflora
Aquilegia atrovinosa
Aquilegia karelinii
Aquilegia moorcroftiana
Aquilegia ecalcarata
Aquilegia oxysepala var. kansuensis
Aquilegia rockii
Aquilegia yabeana
Aquilegia
Aquilegia coerulea
Aquilegia elegantula
Aquilegia longissima
Aquilegia skinneri
Aquilegia chrysantha
Aquilegia flavescens
Aquilegia formosa
Aquilegia eximia
Aquilegia pubescens
Aquilegia scopulorum
Aquilegia canadensis
Aquilegia brevistyla
Aquilegia jonesii
Aquilegia parviflora
Aquilegia amurensis
Aquilegia japonica
Semiaquilegia adoxoides
Enemion biternatum
Paraquilegia grandiflora
Paropyrum anemonoides
Thalictrum minus
0.01

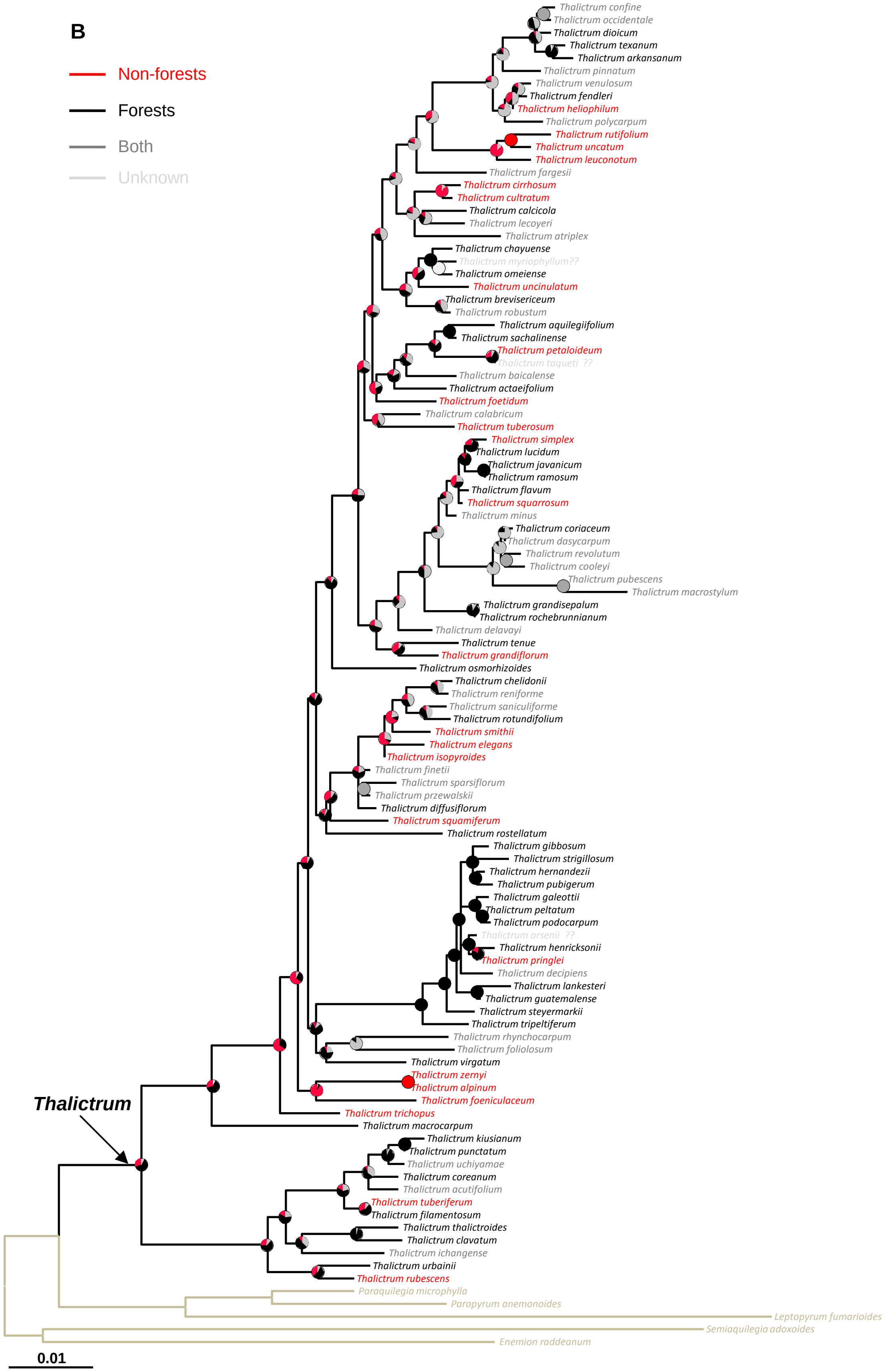
B
Non-forests
Forests
Both
Unknown
Thalictrum confine
Thalictrum occidentale
Thalictrum dioicum
Thalictrum texanum
Thalictrum arkansanum
Thalictrum pinnatum
Thalictrum venulosum
Thalictrum fendleri
Thalictrum heliophilum
Thalictrum polycarpum
Thalictrum rutifolium
Thalictrum uncatum
Thalictrum leuconotum
Thalictrum fargesii
Thalictrum cirrhosum
Thalictrum cultratum
Thalictrum calcicola
Thalictrum lecoyeri
Thalictrum atriplex
Thalictrum chayuense
Thalictrum myriophyllum??
Thalictrum omeiense
Thalictrum uncinulatum
Thalictrum brevisericeum
Thalictrum robustum
Thalictrum aquilegiifolium
Thalictrum sachalinense
Thalictrum petaloideum
Thalictrum taqueti ??
Thalictrum baicalense
Thalictrum actaeifolium
Thalictrum foetidum
Thalictrum calabricum
Thalictrum tuberosum
Thalictrum simplex
Thalictrum lucidum
Thalictrum javanicum
Thalictrum ramosum
Thalictrum flavum
Thalictrum squarrosum
Thalictrum minus
Thalictrum coriaceum
Thalictrum dasycarpum
Thalictrum revolutum
Thalictrum cooleyi
Thalictrum pubescens
Thalictrum macrostylum
Thalictrum grandisepalum
Thalictrum rochebrunnianum
Thalictrum delavayi
Thalictrum tenue
Thalictrum grandiflorum
Thalictrum osmorhizoides
Thalictrum chelidonii
Thalictrum reniforme
Thalictrum saniculiforme
Thalictrum rotundifolium
Thalictrum smithii
Thalictrum elegans
Thalictrum isopyroides
Thalictrum finetii
Thalictrum sparsiflorum
Thalictrum przewalskii
Thalictrum diffusiflorum
Thalictrum squamiferum
Thalictrum rostellatum
Thalictrum gibbosum
Thalictrum strigillosum
Thalictrum hernandezii
Thalictrum pubigerum
Thalictrum galeottii
Thalictrum peltatum
Thalictrum podocarpum
Thalictrum arsenii ??
Thalictrum henricksonii
Thalictrum pringlei
Thalictrum decipiens
Thalictrum lankesteri
Thalictrum guatemalense
Thalictrum steyermarkii
Thalictrum tripeltiferum
Thalictrum rhynchocarpum
Thalictrum foliolosum
Thalictrum virgatum
Thalictrum zernyi
Thalictrum alpinum
Thalictrum foeniculaceum
Thalictrum trichopus
Thalictrum macrocarpum
Thalictrum
Thalictrum kiusianum
Thalictrum punctatum
Thalictrum uchiyamae
Thalictrum coreanum
Thalictrum acutifolium
Thalictrum tuberiferum
Thalictrum filamentosum
Thalictrum thalictroides
Thalictrum clavatum
Thalictrum ichangense
Thalictrum urbainii
Thalictrum rubescens
Paraquilegia microphylla
Paropyrum anemonoides
Leptopyrum fumarioides
Semiaquilegia adoxoides
Enemion raddeanum
0.01

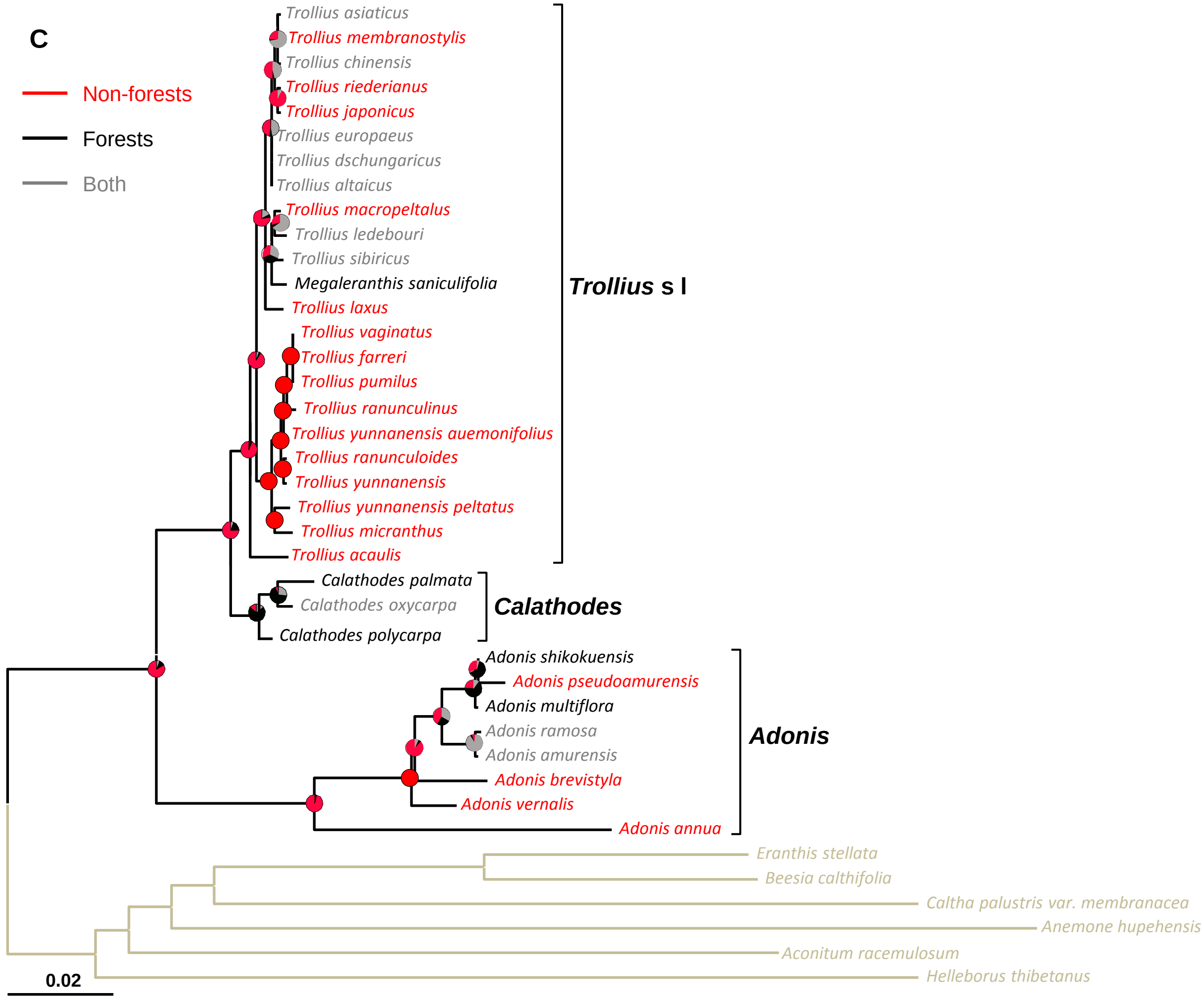
C
Non-forests
Forests
Both
Trollius asiaticus
Trollius membranostylis
Trollius chinensis
Trollius riederianus
Trollius japonicus
Trollius europaeus
Trollius dschungaricus
Trollius altaicus
Trollius macropeltalus
Trollius ledebouri
Trollius sibiricus
Megaleranthis saniculifolia
Trollius laxus
Trollius vaginatus
Trollius farreri
Trollius pumilus
Trollius ranunculinus
Trollius yunnanensis auemonifolius
Trollius ranunculoides
Trollius yunnanensis
Trollius yunnanensis peltatus
Trollius micranthus
Trollius acaulis
Trollius s l
Calathodes palmata
Calathodes oxycarpa
Calathodes polycarpa
Calathodes
Adonis shikokuensis
Adonis pseudoamurensis
Adonis multiflora
Adonis ramosa
Adonis amurensis
Adonis brevistyla
Adonis vernalis
Adonis annua
Adonis
Eranthis stellata
Beesia calthifolia
Caltha palustris var. membranacea
Anemone hupehensis
Aconitum racemulosum
Helleborus thibetanus
0.02

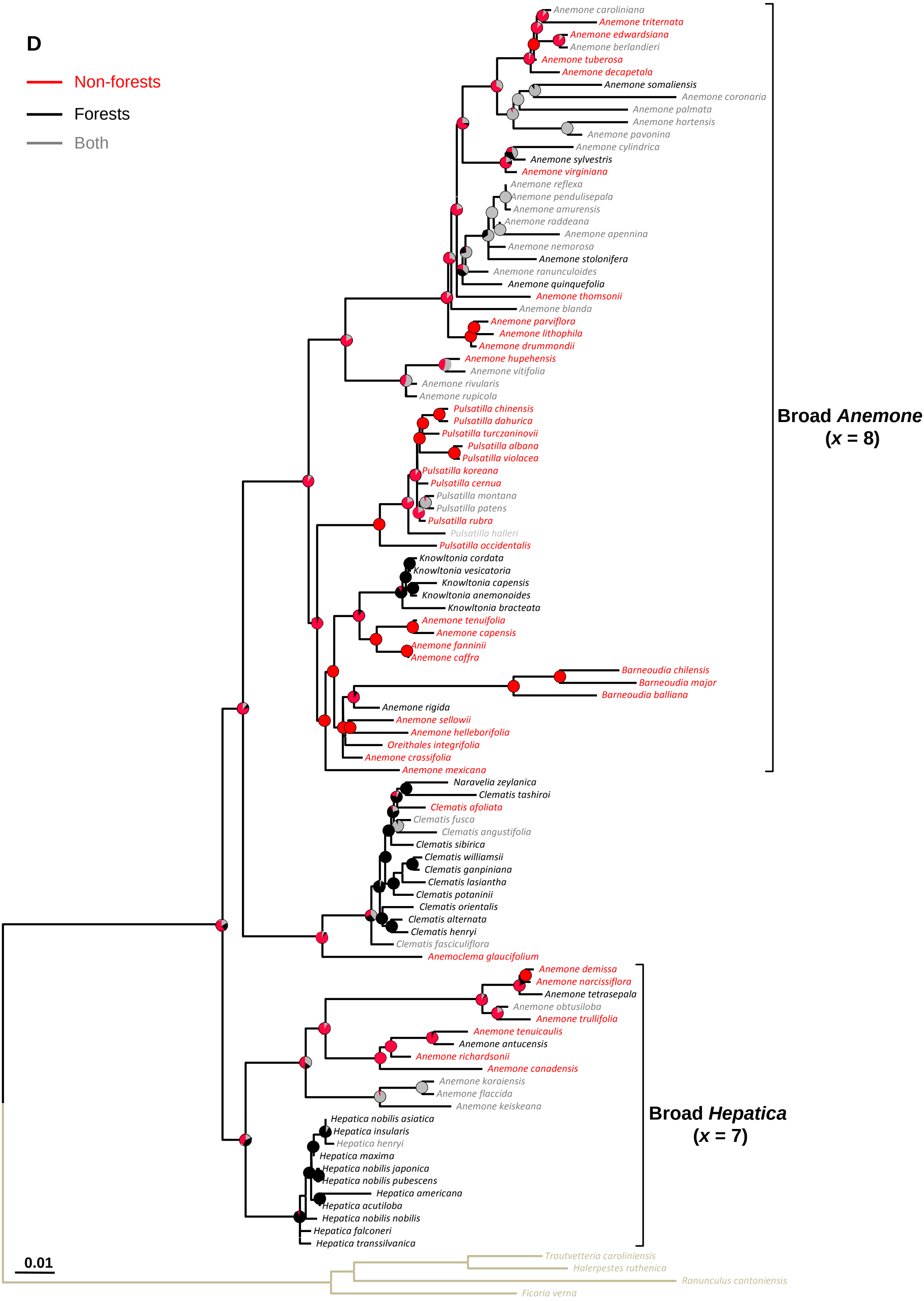

D
Non-forests
Forests
Both
Anemone caroliniana
Anemone triternata
Anemone edwardsiana
Anemone berlandieri
Anemone tuberosa
Anemone decapetala
Anemone somaliensis
Anemone coronaria
Anemone palmata
Anemone hortensis
Anemone pavonina
Anemone cylindrica
Anemone sylvestris
Anemone virginiana
Anemone reflexa
Anemone pendulisepala
Anemone amurensis
Anemone raddeana
Anemone apennina
Anemone nemorosa
Anemone stolonifera
Anemone ranunculoides
Anemone quinquefolia
Anemone thomsonii
Anemone blanda
Anemone parviflora
Anemone lithophila
Anemone drummondii
Anemone hupehensis
Anemone vitifolia
Anemone rivularis
Anemone rupicola
Pulsatilla chinensis
Pulsatilla dahurica
Pulsatilla turczaninovii
Pulsatilla albana
Pulsatilla violacea
Pulsatilla koreana
Pulsatilla cernua
Pulsatilla montana
Pulsatilla patens
Pulsatilla rubra
Pulsatilla halleri
Pulsatilla occidentalis
Knowltonia cordata
Knowltonia vesicatoria
Knowltonia capensis
Knowltonia anemonoides
Knowltonia bracteata
Anemone tenuifolia
Anemone capensis
Anemone fanninii
Anemone caffra
Barneoudia chilensis
Barneoudia major
Barneoudia balliana
Anemone rigida
Anemone sellowii
Anemone helleborifolia
Oreithales integrifolia
Anemone crassifolia
Anemone mexicana
Broad Anemone (x = 8)
Naravelia zeylanica
Clematis tashiroi
Clematis afoliata
Clematis fusca
Clematis angustifolia
Clematis sibirica
Clematis williamsii
Clematis ganpiniana
Clematis lasiantha
Clematis potaninii
Clematis orientalis
Clematis alternata
Clematis henryi
Clematis fasciculiflora
Anemoclema glaucifolium
Anemone demissa
Anemone narcissiflora
Anemone tetrasepala
Anemone obtusiloba
Anemone trullifolia
Anemone tenuicaulis
Anemone antucensis
Anemone richardsonii
Anemone canadensis
Anemone koraiensis
Anemone flaccida
Anemone keiskeana
Hepatica nobilis asiatica
Hepatica insularis
Hepatica henryi
Hepatica maxima
Hepatica nobilis japonica
Hepatica nobilis pubescens
Hepatica americana
Hepatica acutiloba
Hepatica nobilis nobilis
Hepatica falconeri
Hepatica transsilvanica
Broad Hepatica (x = 7)
0.01
Trautvetteria caroliniensis
Halerpestes ruthenica
Ranunculus cantoniensis
Ficaria verna

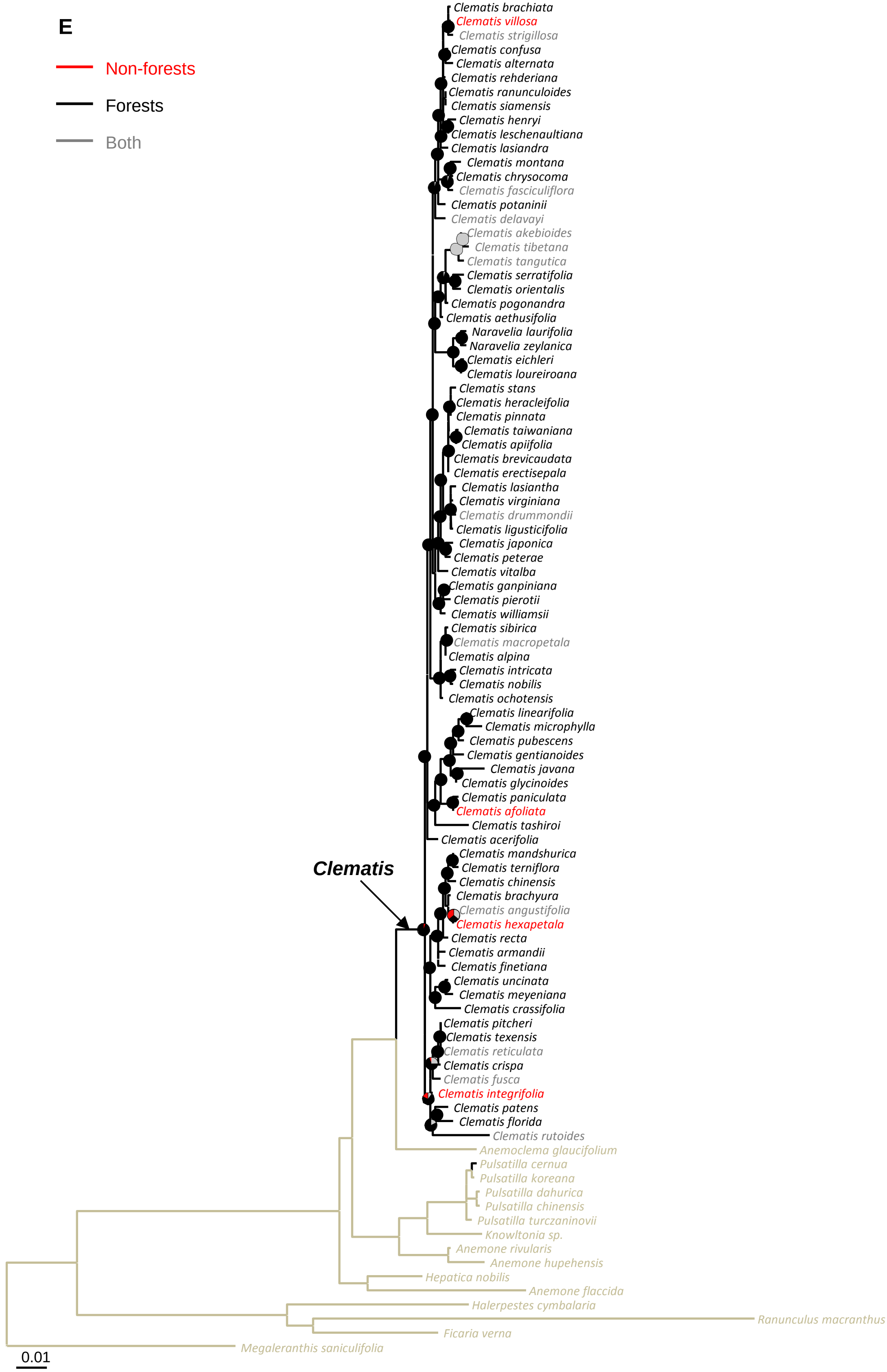

E
Non-forests
Forests
Both
Clematis brachiata
Clematis villosa
Clematis strigillosa
Clematis confusa
Clematis alternata
Clematis rehderiana
Clematis ranunculoides
Clematis siamensis
Clematis henryi
Clematis leschenaultiana
Clematis lasiandra
Clematis montana
Clematis chrysocoma
Clematis fasciculiflora
Clematis potaninii
Clematis delavayi
Clematis akebioides
Clematis tibetana
Clematis tangutica
Clematis serratifolia
Clematis orientalis
Clematis pogonandra
Clematis aethusifolia
Naravelia laurifolia
Naravelia zeylanica
Clematis eichleri
Clematis loureiroana
Clematis stans
Clematis heracleifolia
Clematis pinnata
Clematis taiwaniana
Clematis apiifolia
Clematis brevicaudata
Clematis erectisepala
Clematis lasiantha
Clematis virginiana
Clematis drummondii
Clematis ligusticifolia
Clematis japonica
Clematis peterae
Clematis vitalba
Clematis ganpiniana
Clematis pierotii
Clematis williamsii
Clematis sibirica
Clematis macropetala
Clematis alpina
Clematis intricata
Clematis nobilis
Clematis ochotensis
Clematis linearifolia
Clematis microphylla
Clematis pubescens
Clematis gentianoides
Clematis javana
Clematis glycinoides
Clematis paniculata
Clematis afoliata
Clematis tashiroi
Clematis acerifolia
Clematis
Clematis mandshurica
Clematis terniflora
Clematis chinensis
Clematis brachyura
Clematis angustifolia
Clematis hexapetala
Clematis recta
Clematis armandii
Clematis finetiana
Clematis uncinata
Clematis meyeniana
Clematis crassifolia
Clematis pitcheri
Clematis texensis
Clematis reticulata
Clematis crispa
Clematis fusca
Clematis integrifolia
Clematis patens
Clematis florida
Clematis rutoides
Anemoclema glaucifolium
Pulsatilla cernua
Pulsatilla koreana
Pulsatilla dahurica
Pulsatilla chinensis
Pulsatilla turczaninovii
Knowltonia sp.
Anemone rivularis
Anemone hupehensis
Hepatica nobilis
Anemone flaccida
Halerpestes cymbalaria
Ranunculus macranthus
Ficaria verna
Megaleranthis saniculifolia
0.01

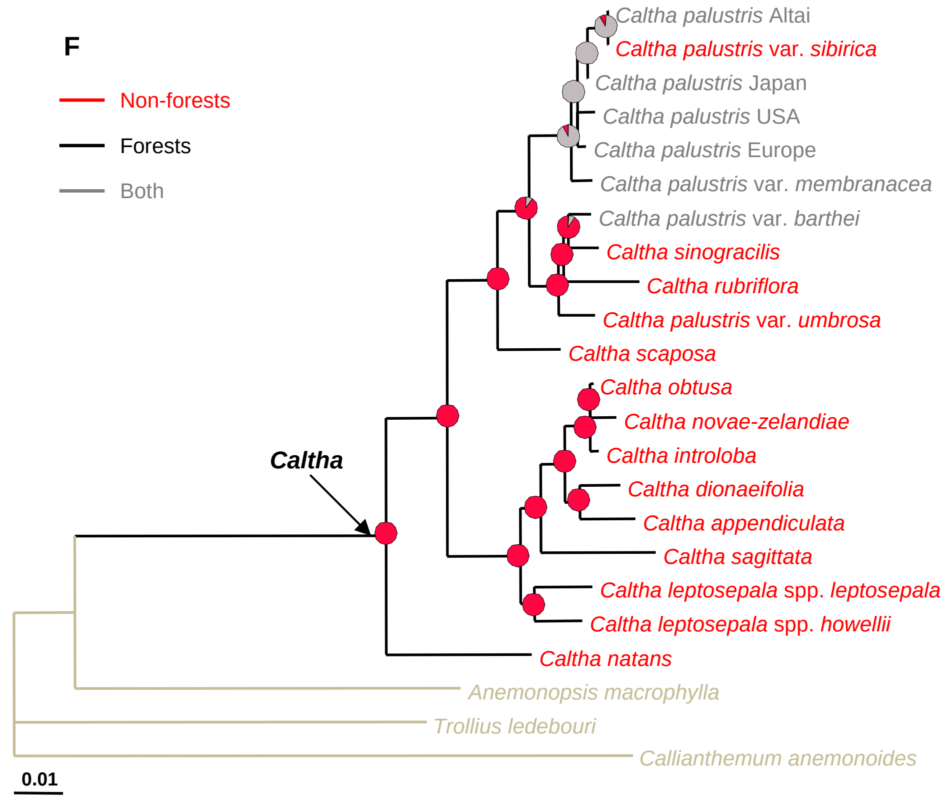
F
Non-forests
Forests
Both
Caltha palustris Altai
Caltha palustris var. sibirica
Caltha palustris Japan
Caltha palustris USA
Caltha palustris Europe
Caltha palustris var. membranacea
Caltha palustris var. barthei
Caltha sinogracilis
Caltha rubriflora
Caltha palustris var. umbrosa
Caltha scaposa
Caltha obtusa
Caltha novae-zelandiae
Caltha introloba
Caltha dionaeifolia
Caltha appendiculata
Caltha sagittata
Caltha leptosepala spp. leptosepala
Caltha leptosepala spp. howellii
Caltha natans
Caltha
Anemonopsis macrophylla
Trollius ledebouri
Callianthemum anemonoides
0.01

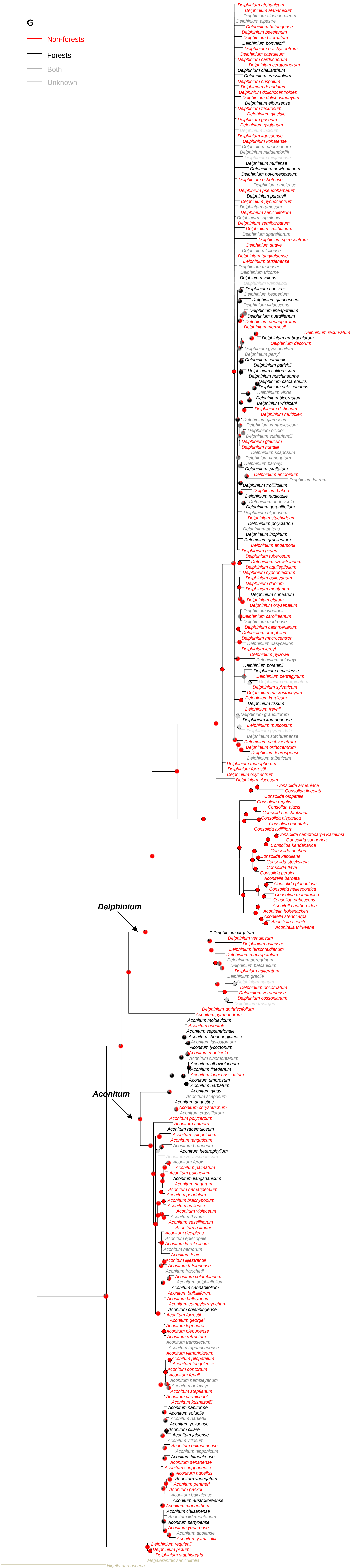
G
Non-forests
Forests
Both
Unknown
Delphinium
Aconitum
Delphinium afghanicum
Delphinium alabamicum
Delphinium albocoeruleum
Delphinium alpestre
Delphinium batangense
Delphinium beesianum
Delphinium biternatum
Delphinium bonvalotii
Delphinium brachycentrum
Delphinium caeruleum
Delphinium carduchorum
Delphinium ceratophorum
Delphinium cheilanthum
Delphinium crassifolium
Delphinium crispulum
Delphinium denudatum
Delphinium dolichocentroides
Delphinium dolichostachyum
Delphinium elbursense
Delphinium flexuosum
Delphinium glaciale
Delphinium griseum
Delphinium gyalanum
Delphinium kansuense
Delphinium kohatense
Delphinium maackianum
Delphinium middendorffii
Delphinium muliense
Delphinium newtonianum
Delphinium novomexicanum
Delphinium ochotense
Delphinium omeiense
Delphinium pseudohamatum
Delphinium purpusii
Delphinium pycnocentrum
Delphinium ramosum
Delphinium saniculifolium
Delphinium sapellonis
Delphinium semibarbatum
Delphinium smithianum
Delphinium sparsiflorum
Delphinium spirocentrum
Delphinium suave
Delphinium taliense
Delphinium tangkulaense
Delphinium tatsienense
Delphinium treleasei
Delphinium tricorne
Delphinium valens
Delphinium hansenii
Delphinium hesperium
Delphinium viridescens
Delphinium glaucescens
Delphinium lineapetalum
Delphinium nuttallianum
Delphinium depauperatum
Delphinium menziesii
Delphinium recurvatum
Delphinium umbraculorum
Delphinium decorum
Delphinium gypsophilum
Delphinium parryi
Delphinium cardinale
Delphinium parishii
Delphinium californicum
Delphinium hutchinsonae
Delphinium calcarequitis
Delphinium subscandens
Delphinium viride
Delphinium bicornutum
Delphinium wislizeni
Delphinium distichum
Delphinium multiplex
Delphinium glareosum
Delphinium xantholeucum
Delphinium bicolor
Delphinium sutherlandii
Delphinium glaucum
Delphinium nuttallii
Delphinium scaposum
Delphinium variegatum
Delphinium barbeyi
Delphinium exaltatum
Delphinium antoninum
Delphinium luteum
Delphinium trolliifolium
Delphinium bakeri
Delphinium nudicaule
Delphinium andesicola
Delphinium geraniifolium
Delphinium uliginosum
Delphinium stachydeum
Delphinium polycladon
Delphinium patens
Delphinium inopinum
Delphinium gracilentum
Delphinium andersonii
Delphinium geyeri
Delphinium tuberosum
Delphinium szowitsianum
Delphinium aquilegifolium
Delphinium cyphoplectrum
Delphinium bulleyanum
Delphinium dubium
Delphinium montanum
Delphinium cuneatum
Delphinium elatum
Delphinium oxysepalum
Delphinium wootonii
Delphinium carolinianum
Delphinium madrense
Delphinium cashmerianum
Delphinium oreophilum
Delphinium macrocentron
Delphinium dasycaulon
Delphinium leroyi
Delphinium pylzowii
Delphinium delavayi
Delphinium potaninii
Delphinium nevadense
Delphinium pentagynum
Delphinium sylvaticum
Delphinium macrostachyum
Delphinium kurdicum
Delphinium fissum
Delphinium freynii
Delphinium grandiflorum
Delphinium kamaonense
Delphinium muscosum
Delphinium sutchuenense
Delphinium pachycentrum
Delphinium orthocentrum
Delphinium tsarongense
Delphinium thibeticum
Delphinium trichophorum
Delphinium forrestii
Delphinium oxycentrum
Delphinium viscosum
Consolida armeniaca
Consolida lineolata
Consolida olopetala
Consolida regalis
Consolida ajacis
Consolida uechtritziana
Consolida hispanica
Consolida orientalis
Consolida axilliflora
Consolida camptocarpa Kazakhst
Consolida songorica
Consolida kandaharica
Consolida aucheri
Consolida kabuliana
Consolida stocksiana
Consolida flava
Consolida persica
Aconitella barbata
Consolida glandulosa
Consolida hellespontica
Consolida mauritanica
Consolida pubescens
Aconitella anthoroidea
Aconitella hohenackeri
Aconitella stenocarpa
Aconitella aconiti
Aconitella thirkeana
Delphinium virgatum
Delphinium venulosum
Delphinium balansae
Delphinium hirschfeldianum
Delphinium macropetalum
Delphinium peregrinum
Delphinium balcanicum
Delphinium halteratum
Delphinium gracile
Delphinium obcordatum
Delphinium verdunense
Delphinium cossonianum
Delphinium anthriscifolium
Aconitum gymnandrum
Aconitum moldavicum
Aconitum orientale
Aconitum septentrionale
Aconitum shennongjiaense
Aconitum lasiostomum
Aconitum lycoctonum
Aconitum monticola
Aconitum sinomontanum
Aconitum alboviolaceum
Aconitum finetianum
Aconitum longecassidatum
Aconitum umbrosum
Aconitum barbatum
Aconitum gigas
Aconitum scaposum
Aconitum angustius
Aconitum chrysotrichum
Aconitum crassiflorum
Aconitum polycarpum
Aconitum anthora
Aconitum racemulosum
Aconitum spiripetalum
Aconitum tanguticum
Aconitum brunneum
Aconitum heterophyllum
Aconitum ferox
Aconitum palmatum
Aconitum pulchellum
Aconitum liangshanicum
Aconitum nagarum
Aconitum hamatipetalum
Aconitum pendulum
Aconitum brachypodum
Aconitum hulilense
Aconitum violaceum
Aconitum flavum
Aconitum sessiliflorum
Aconitum balfourii
Aconitum decipiens
Aconitum episcopale
Aconitum karakolicum
Aconitum nemorum
Aconitum tsaii
Aconitum lijestrandii
Aconitum tatsienense
Aconitum franchetii
Aconitum columbianum
Aconitum delphinifolium
Aconitum cannabifolium
Aconitum bulbilliferum
Aconitum bulleyanum
Aconitum campylorrhynchum
Aconitum chiennigense
Aconitum forrestii
Aconitum georgei
Aconitum legendrei
Aconitum piepunense
Aconitum refractum
Aconitum transsectum
Aconitum tuguancunense
Aconitum vilmorinianum
Aconitum pilopetalum
Aconitum tongolense
Aconitum contortum
Aconitum fengii
Aconitum hemsleyanum
Aconitum delavayi
Aconitum stapfianum
Aconitum carmichaelii
Aconitum kusnezoffii
Aconitum napiforme
Aconitum volubile
Aconitum bartlettii
Aconitum yezoense
Aconitum ciliare
Aconitum jaluense
Aconitum villosum
Aconitum hakusanense
Aconitum nipponicum
Aconitum kitadakense
Aconitum senanense
Aconitum sungpanense
Aconitum napellus
Aconitum variegatum
Aconitum pentheri
Aconitum paskoi
Aconitum baicalense
Aconitum austrokoreense
Aconitum monanthum
Aconitum chiisanense
Aconitum iidemontanum
Aconitum sanyoense
Aconitum yuparense
Aconitum apoiense
Aconitum yamazakii
Delphinium requienii
Delphinium pictum
Delphinium staphisagria
Megaleranthis saniculifolia
Nigella damascena

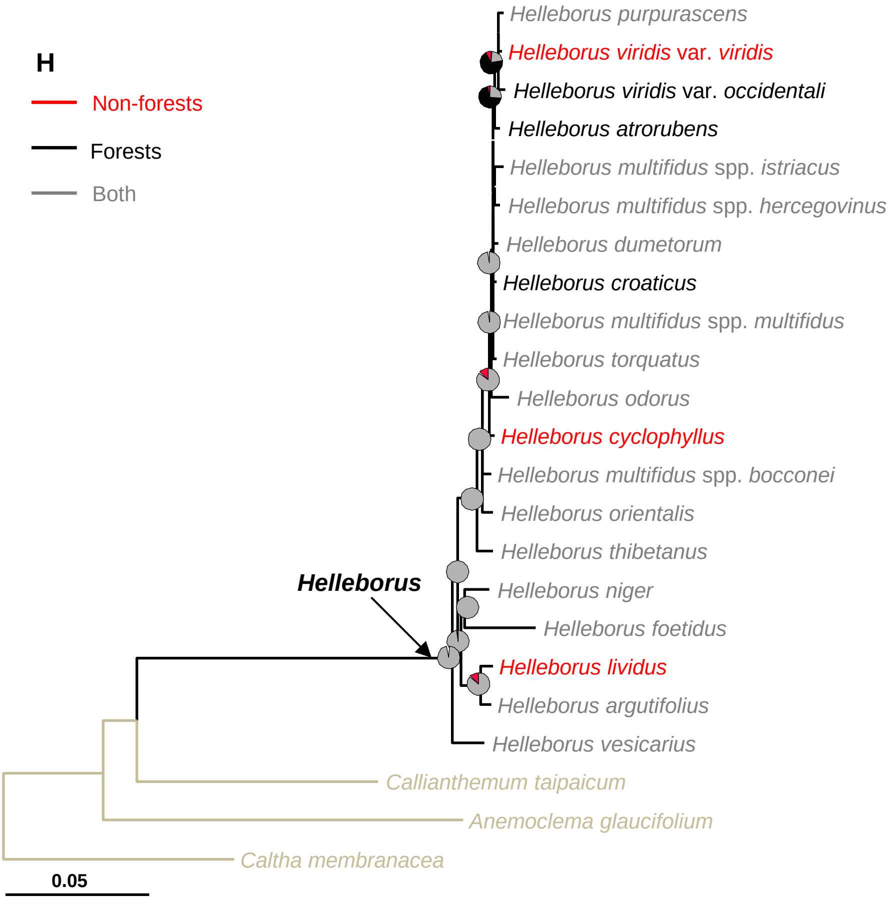
H
Non-forests
Forests
Both
Helleborus purpurascens
Helleborus viridis var. viridis
Helleborus viridis var. occidentali
Helleborus atrorubens
Helleborus multifidus spp. istriacus
Helleborus multifidus spp. hercegovinus
Helleborus dumetorum
Helleborus croaticus
Helleborus multifidus spp. multifidus
Helleborus torquatus
Helleborus odorus
Helleborus cyclophyllus
Helleborus multifidus spp. bocconei
Helleborus orientalis
Helleborus thibetanus
Helleborus
Helleborus niger
Helleborus foetidus
Helleborus lividus
Helleborus argutifolius
Helleborus vesicarius
Callianthemum taipaicum
Anemoclema glaucifolium
Caltha membranacea
0.05

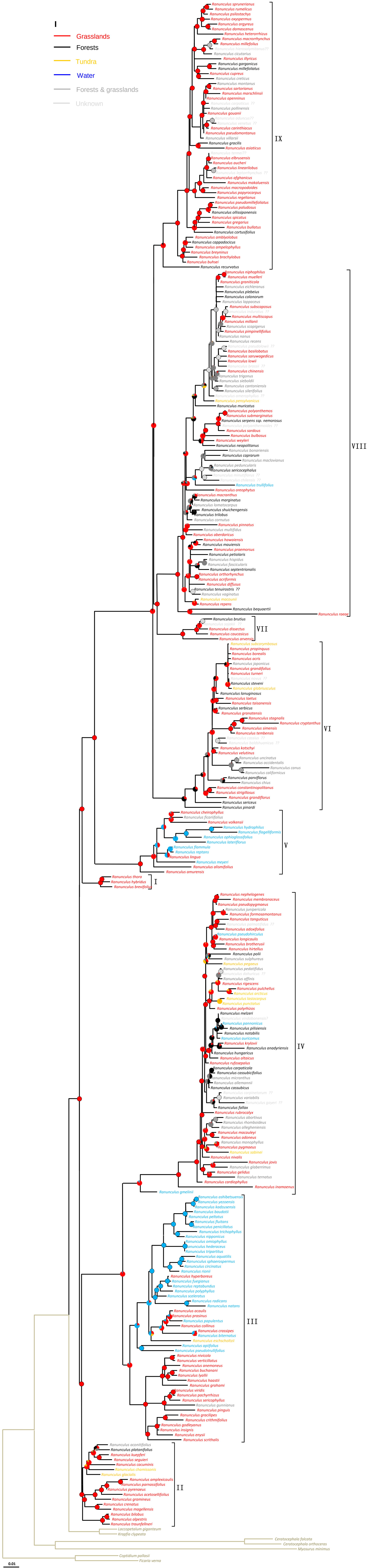
I
Grasslands
Forests
Tundra
Water
Forests & grasslands
Unknown
IX
VIII
VII
VI
V
I
IV
III
II
0.01
